# A novel whole cancer cell vaccine based on modified β-glucan elicits robust anti-tumor immunity

**DOI:** 10.7150/thno.121911

**Published:** 2026-01-01

**Authors:** Jianhan Huang, Yuyuan Wang, Junrong Zhu, Li Li, Lang Hu, Yuan Zhou, Baoguo Xiao, Yao Yu

**Affiliations:** 1 Department of Neurosurgery, Huashan Hospital, Fudan University, Shanghai, 200000, PR China.; 2 National Center for Neurological Disorders, Fudan University, Shanghai, 200000, PR China.; 3 Shanghai Key Laboratory of Brain Function and Restoration and Neural Regeneration, Fudan University, Shanghai, 200000, PR China.; 4 Immunology Laboratory, Neurosurgical Institute of Fudan University, Fudan University, Shanghai, 200000, PR China.; 5 Shanghai Clinical Medical Center of Neurosurgery, Fudan University, Shanghai, 200000, PR China.; 6 Department of Dermatology, Huashan Hospital, Fudan University, Shanghai, 200000, PR China.; 7 Department of Neurology, Huashan Hospital, Fudan University, Shanghai, 200000, PR China.; 8 Hubei Provincial Hospital of Traditional Chinese Medicine (Affiliated Hospital of Hubei University of Chinese Medicine, Hubei Provincial Academy of Traditional Chinese Medicine, Hubei Shizhen Laboratory), Wuhan, 430061, PR China.

**Keywords:** glioblastoma, cancer vaccine, β-glucan, immunotherapy, whole cancer cell vaccine

## Abstract

**Background**: Although autologous whole tumour cells provide broad-spectrum antigens for personalised cancer vaccines, their weak immunogenicity necessitates adjuvant co-delivery systems.

**Methods**: We developed a conjugate adjuvant (G-PL) by coupling modified yeast β-glucan with poly-D-lysine. Electron microscopy confirmed its binding to GL261 cell membranes. The adjuvant-cell complex (ICC@G-PL) was constructed by coating irradiated tumour cells with G-PL. We evaluated the recruitment/activation of dendritic cells (DCs), lymph node priming, tumour-specific immunity, and therapeutic efficacy in glioblastoma, colon cancer, and melanoma models. Dectin-1-mediated Th17 induction was analysed via Western blotting and flow cytometry.

**Results**: G-PL (≤ 500 μg/mL) rapidly adhered to cell membranes without cytotoxicity. *In vitro*, it enhanced DC uptake of tumour components, maturation, and non-pathogenic Th17 differentiation. *In vivo*, ICC@G-PL recruited DCs at injection sites, activated draining lymph nodes, and elevated plasma levels of IL-12, TNF-α, and IFN-γ. The vaccine prolonged survival in both therapeutic and preventive models, increasing intratumoral CD8^+^/CD4^+^ T cell ratios, M1 macrophages, and neutrophils. Dectin-1 downregulation in DCs correlated with Th17-driven anti-tumour responses.

**Conclusions**: G-PL, a novel β-glucan-based adjuvant, enables rapid construction of autologous whole-cell vaccines. This strategy enhances tumour-specific immunity and reprogrammes the tumour microenvironment, offering a universal platform for personalised cancer immunotherapy.

## Introduction

Recent advances in cancer immunotherapy—such as the success of chimeric antigen receptor T cell (CAR-T) and immune checkpoint blocker therapies—have highlighted the transformative potential of harnessing the immune system to treat cancer 1, 2. Among these strategies, cancer vaccines remain compelling as they directly target tumour-specific antigens (TSAs) or tumour-associated antigens (TAAs) to activate adaptive immunity. Since the identification of these antigens, vaccines utilising tumour lysates, purified peptides, nucleic acids, or inactivated cancer cells have been explored. Autologous tumour cells (ATCs) have emerged as a unique platform due to their inherent ability to encapsulate the full spectrum of tumour antigens without prior neoantigen identification, along with their distinct cellular physical properties 3-5.

ATC-based vaccines (also termed whole tumour cell vaccines, WCCVs) leverage the patient's own resected or biopsied tumour tissue, naturally incorporating both common TSA/TAA and personalised immunogenic neoantigens. This approach overcomes the limitation of antigen heterogeneity that affects single-antigen or predefined multi-antigen vaccines 6. Theoretically, this pan-antigen coverage enables personalised immune activation tailored to each patient's unique tumour profile, minimising the risk of immune evasion caused by antigen loss 3. Clinically, ATCs are typically inactivated by radiation or mitomycin to eliminate proliferative capacity while preserving antigenicity, and are often combined with adjuvants such as granulocyte-macrophage colony-stimulating factor (GM-CSF) or interleukins to enhance antigen presentation by dendritic cells (DCs) 7. Preclinical studies have demonstrated their potential to induce tumour-specific T cell responses in models of melanoma, renal cell carcinoma, and prostate cancer, with early-phase clinical trials reporting acceptable safety profiles and isolated cases of durable remission 8, 9.

However, ATC vaccines face critical limitations hindering their potential for clinical translation. First, preparation complexity remains a major barrier: current protocols require fresh tumour tissue, *ex vivo* gene editing for adjuvant integration, and prolonged manufacturing timelines of up to 2-4 weeks or longer, thereby delaying their administration during the early postoperative period when immune intervention is most critical 10. Second, intrinsic immunogenicity challenges persist—many ATCs exhibit poor antigen presentation due to inherent MHC/antigen-processing defects or activation of immune escape pathways such as downregulation of co-stimulatory molecules and upregulation of PD-L1. This limits their ability to effectively activate DCs and downstream T cells even when combined with conventional adjuvants 11. Third, the therapeutic efficacy of traditional ATC vaccines is modest, with most clinical trials reporting objective response rates of only 10-20%, underscoring the need for innovative strategies to enhance their immunostimulatory capacity 12.

Simplified yet more immunogenic ATC vaccine platforms are urgently needed. Recent advances in chemical engineering—such as single-cell encapsulation—offer a feasible approach to rapidly construct co-delivery systems without relying on time-consuming gene editing, enabling the incorporation of diverse adjuvants (including non-protein molecules) for immediate immune activation 13. In this context, yeast-derived β-glucan has emerged as a promising adjuvant candidate due to its potent immune-stimulating properties, including DC activation via Dectin-1 and complement receptors, and promotion of pro-inflammatory cytokine release. Building on this, we developed a fast cell-adhering adjuvant, G-PL, based on yeast-derived β-glucan and constructed a novel WCCV. Our results demonstrate that this personalised strategy successfully induces tumour-specific T cells and achieves desirable therapeutic efficacy across multiple solid tumour types, while also elucidating the unique interactions between soluble β-glucan and DCs.

## Methods

**Materials:** Yeast-derived soluble β-glucan and poly-D-polylysine were obtained from Solarbio Life Sciences, China. Dulbecco's modified Eagle medium (DMEM), Roswell Park Memorial Institute medium (RPMI) 1640, and fetal bovine serum (FBS) were obtained from Gibco, USA. Phosphate-buffered saline (PBS) and penicillin/streptomycin were obtained from HyClone Logan, USA. Other reagents include GL261-luciferase, U87, CT26, B16 cells (West China Hospital); Raw264.7 (Procell Biotechnology, China); GM-CSF, IL-4 (Novel Protein, China); LPS, DNAase, collagenase IV (Sangon Biotechnology, China); and antibodies/assay kits (BD Biosciences, R&D Systems, Biolegend, Proteintech, USA).

**Cell culture:** Cancer cell lines were cultured in DMEM, while Raw264.7 cells were cultured in RPMI 1640. All the media contained 10% FBS and 1% penicillin/streptomycin at 37°C with 5% CO₂.

**G-PL synthesis:** Carboxyl β-glucan was synthesised via DMAP-catalysed esterification of β-glucan (0.07 g/mL) with butanedioic anhydride (0.31 g/mL in dimethyl sulfoxide) at 60 °C for 16 h at a stirring speed of 300 rpm. Post-dialysis (15 kDa molecular weight cut-off [MWCO]) and lyophilisation, G-PL was stored at -80 °C.

**G-PL characterisation:** The structure was confirmed by proton nuclear magnetic resonance (¹H-NMR, Varian 400 MHz), and the particle size/zeta potential were analysed via Zeta View (Particle Metrix). Cellular uptake was assessed using G-PL-fluorescein isothiocyanate (FITC)-treated GL261 cells (1×10⁶ cells/mL) analysed by flow cytometry. Cell morphology was examined via scanning and transmission electron microscopy (SEM, Regulus 8100; TEM, HT7800) after fixation with 4% paraformaldehyde (PFA)/0.5% glutaraldehyde.

**Preparation of G-PL-FITC-treated GL261 cells:** The preparation involved 1 mg/mL G-PL labelled with FITC at a concentration of 100 μg/mL under 4 °C for 2 h. The free FITC was removed by two overnight dialyses (15 kDa MWCO). GL261 cells (1×10^6^/mL) were labelled with 500 μg/mL G-PL-FITC at room temperature for 20 min for subsequent experiments.

**Apoptosis assay:** GL261 cells (1×10⁵/well) were treated with G-PL for 48 h, stained with 7-AAD, and analysed by flow cytometry.

**Bone marrow-derived DC (BMDC) Differentiation:** BM cells from C57BL6 mice purchased from Shanghai Model Organisms were cultured in RPMI 1640 with 20 ng/mL GM-CSF and 10 ng/mL IL-4 for 5 days. The media were half-refreshed every 48 h. Loosely adherent cells were harvested as immature BMDCs.

***In vitro* uptake/activation:** CFSE-labelled GL261 cells ± G-PL or soluble β-glucan were co-cultured with Cytotell Orange-labelled BMDCs (1:1 ratio) for 2 h. The uptake was monitored via confocal microscopy (FV4000) and flow cytometry. DC maturation (CD80/CD86/IFN-β/TNF-α) was assessed after a 48-hour co-culture with PBS, GL261, or GL261^+^β-glucan. Splenocytes (1×10⁶) were added post-activation for T cell analysis.

**Specimen preparation for Syk-Erk and Dectin-1 analysis:** BMDCs (2×10⁶) were co-cultured with GL261 (1×10⁶) ± G-PL/β-glucan. CD11c⁺ cells were isolated via magnetic beads at 5 min-6 h intervals. The BMDC cells in the samples were separated from the co-culture using anti-CD11c-coated magnetic beads (Miltenyi, Germany) at 5 min, 30 min, 1 h, 2 h, 4 h, and 6 h after co-culture. The DCs that were collected were stained with PE-dectin-1 (Biolegend, USA) for flow cytometry or lysed for western blot (WB).

***In vitro* T cell activation:** C57BL6 splenocytes were filtered (70 μm) and co-cultured with DCs (DC:T=1:10). CD3⁺ T cells were isolated using immunomagnetic beads (Miltenyi). IL-2 was measured via enzyme-linked immunosorbent assay (ELISA); proliferation (day 2), RNA-seq (day 3), and Th17 differentiation (day 5 + Monensin) were assessed.

***In vivo* DC recruitment:** G-PL-coated irradiated GL261 cells (1×10⁶) were injected into mouse footpads (50 µL). After 48 h, CD11c⁺ DC migration was tracked via confocal/*in vivo* imaging system (IVIS; Caliper Life Sciences) imaging. Antigen transfer to the lymph nodes was monitored using CFSE/DID-labelled cells.

**Lymph node activation:** Popliteal lymph nodes were harvested 7 days post-vaccination. Frozen sections were stained with GL-7-antigen-presenting cells (APC)/DAPI, and the germinal centre size was quantified via confocal microscopy.

**Serum cytokines:** Inflammatory markers were analysed using the BD Cytometric Bead Array. The concentrations of the markers were calculated against standard curves (detection limits: 10-2500 pg/mL).

**T cell response:** C57BL6 mice were vaccinated s.c. with PBS, WCCV, WCCV+β-glucan, or G-PL-WCCV (days 1/7). Splenocytes were harvested on day 9 and co-cultured with GL261 for IFN-γ ELISPOT. GL261-specific CD8⁺ T cells were analysed via intracellular flow cytometry (50:1 E:T ratio + Monensin).

**Immunisation:** C57BL/6J and BALB/C purchased from Shanghai Model Organisms mice were vaccinated biweekly with PBS, WCCV, WCCV+β-glucan, or G-PL-WCCV (CT26 cells for BALB/C). The sera were collected 3 days post-vaccination for immunoglobulin (Ig)G isotyping (ELISA against GL261 lysates).

**Bioluminescence:** The tumour growth was monitored via IVIS 10 min post-D-luciferin injection (150 mg/kg i.p.).

**Xenograft model:** GL261-luc cells (2×10⁵) were injected intracranially (2 mm lateral, 1 mm anterior to bregma; 3 mm depth). Tumour establishment was confirmed via bioluminescence on day 5.

**TME analysis:** Tumours were digested with collagenase IV/DNAase on day 20. Single-cell suspensions were stained with FVS620 and antibodies (CD45, CD11b, Ly6G/C, CD3/4/8, PD-1, F4/80, CD86/206). The M1/M2 ratios were determined via Cytoflex S (Beckman).

**Statistics:** Data were analysed using GraphPad Prism v9.5. Significance was determined using unpaired t-tests, one-way analysis of variance with Bonferroni's post-hoc correction for multiple comparisons (*p < 0.05, **p < 0.01, ***p < 0.001, ****p < 0.0001), or log-rank tests (survival).

## Results

### Characterisation and cancer cell encapsulation capability of novel adjuvant G-PL

**Figure 1:**
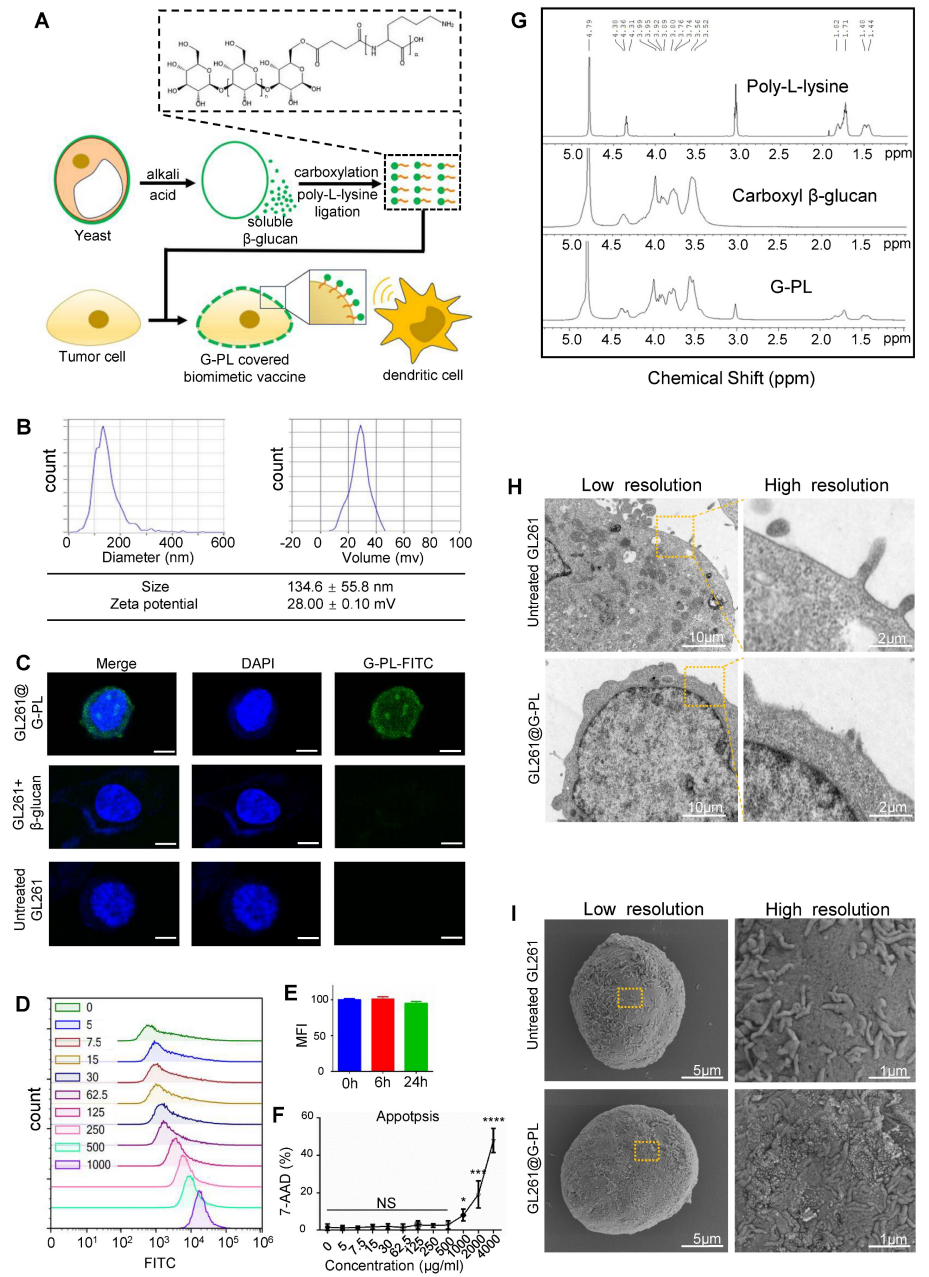
** Characterisation and cancer cell encapsulation capability of the novel adjuvant (G-PL system) (A)** Schematic diagram of the preparation and cell attachment of G-PL. **(B)** Size distribution and Zeta potential of G-PL; data are presented as mean±standard deviation (SD). **(C)** Confocal monitoring of the attachment of G-PL to the GL261 membrane. In the G-PL@GL261 group, GL261 cells were incubated with 500 μg/ml FITC-labelled G-PL for 20 min. In the GL261+β-glucan group, the G-PL was replaced with soluble FITC-labelled β-glucan. In the untreated GL261 group, the GL261 cells were maintained in PBS solution for 20 min before microscopy. Scale bars: 5 μm. **(D)** Flow cytometry analysis of G-PL loading on GL261 cells incubated with different concentrations of G-PL-FITC solution (μg/mL).** (E)** Mean fluorescence intensity (MFI; mean±SD) of GL261 incubated with 500 μg/mL G-PL at different time points, compared with the primary MFI after incubation. **(F)** Cytotoxicity of G-PL to the GL261 cells after 48-h incubation. **(G)** ^1^H-NMR analysis of the characteristic peaks of G-PL, β-glucan, and poly-lysine. **(H)** TEM image of G-PL-encapsulated cells compared with the original GL261 cells. Scale bars separately represent 10 μm and 2 μm in the low- and high-resolution images, respectively. **(I)** SEM image of G-PL-encapsulated cells compared with the original GL261 cells. Scale bars separately represent 5 μm and 1 μm in the low- and high-resolution images, respectively.

We designed a compound named G-PL by conjugating yeast-derived soluble β-glucan with poly-D-lysine (**Figure 1A, S1A**). β-glucan was first carboxylated via DMAP-catalysed esterification with butanedioic anhydride to introduce carboxyl groups into its skeleton, enabling its conjugation to the amine groups of poly-lysine through amidation. Successful coupling was verified by ^1^H-NMR (**Figure 1G**), which showed characteristic peaks of both β-glucan and poly-lysine in the G-PL conjugate. As expected, G-PL was positively charged with a zeta potential of 28±0.1 mV (**Figure 1B**); it was 134.6±55.8 nm in diameter, similar to unmodified soluble β-glucan (**Figure S1C**). This modification enabled G-PL to attach to negatively charged cell membranes. We next labelled G-PL with FITC to track its localisation and incubated it with murine glioblastoma GL261 cells. Confocal laser scanning microscopy (CLSM) showed FITC fluorescence predominantly on the cancer cell surface (**Figure 1C**). Flow cytometry revealed homogeneous G-PL-FITC coating forming a single peak, with the fluorescence intensity (representing adjuvant loading) correlating with incubation concentration (**Figure 1D**), and persisting for over 24 h (**Figure 1E**). GL261 cells incubated with serially diluted G-PL for 24 h at 37°C showed minimal cytotoxicity until the concentration reached 1 mg/mL, as assessed by 7-AAD staining (**Figure 1F**). Acute cytotoxicity for human cells were tested by haemolytic assay using red blood cells (RBCs) incubated with serial G-PL dilutions for 1 h at 37 °C, yielding similar results (**Figure S1D**). SEM and TEM of G-PL-coated cells revealed substance accumulation on the outer layer of the cell membrane, creating a rougher surface without compromising membrane integrity or cell morphology (**Figure 1H-I**). Together, these results verify the synthesis, cell attachment properties, and low cytotoxicity of G-PL. Since 500 μg/mL was the highest non-cytotoxic concentration, cell encapsulation was standardised using 1×10^6^ cells per mL of 500 μg/mL G-PL solution.

### Antigen uptake and APC activation prompted by G-PL

**Figure 2:**
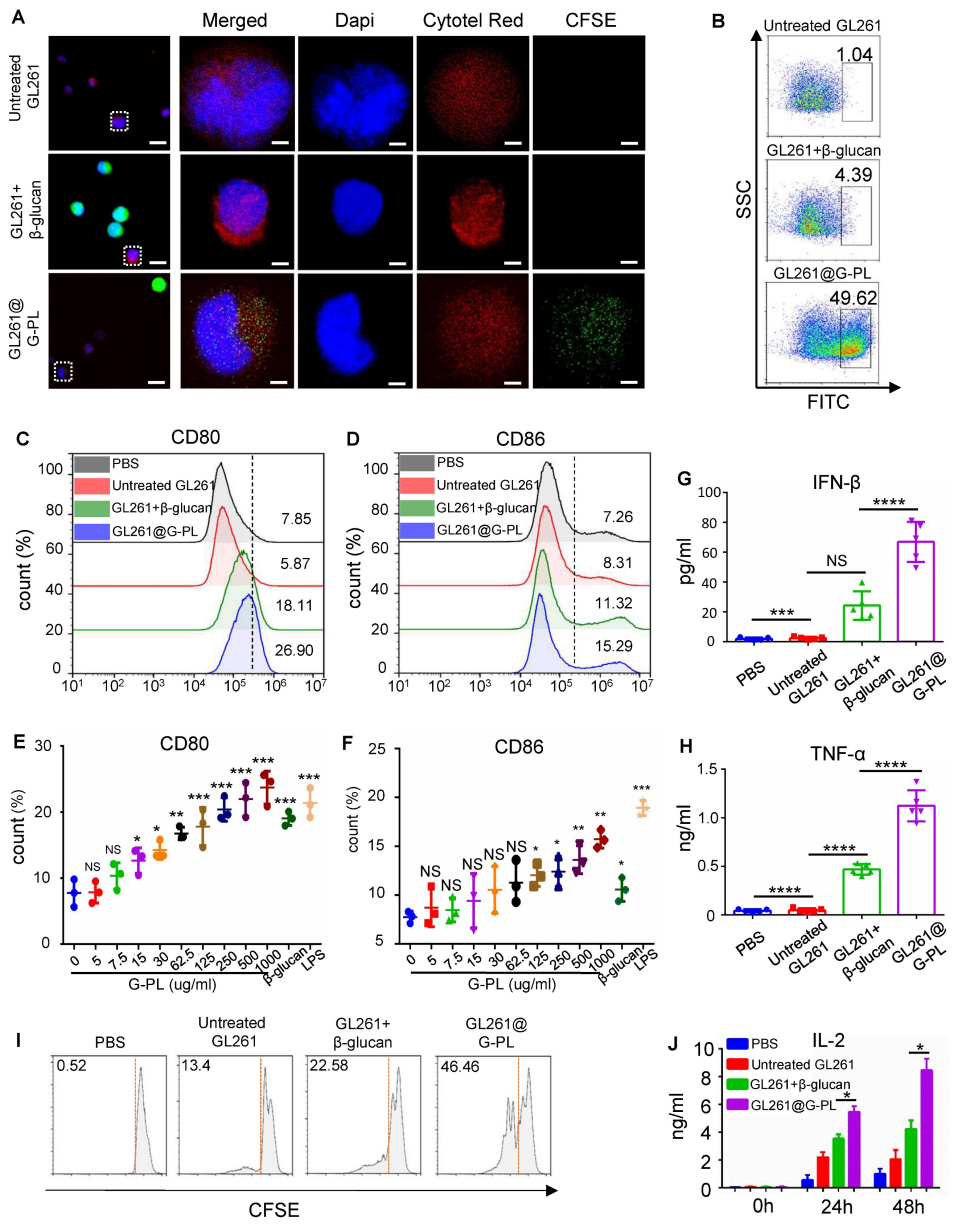
** Antigen uptake and APC activation prompted by G-PL (A)** Confocal imaging of increased CFSE-labelled GL261 uptake by BMDCs after 2 h co-culture; intrinsic GL261 (blank, no adjuvant) and GL261 with soluble β-glucan were used as control. The nucleus is marked by DAPI, while the cytoplasm of GL261 and BMDC cells is separately marked by the cell-probe CFSE and Cytotell Red. Scale bar, 5 μm. **(B)** Flow cytometry analysis of the number of BMDCs undergoing antigen uptake after co-culture. **(C) and (D)** BMDC maturation after 48 h co-culture with GL261 cells that were incubated with 500 μg/ml G-PL for 20 min. The gating strategy is based on the fluorescent signal of DC stained with control IgG. **(E)** and **(F)** BMDC maturation after 48 h co-culture with GL261 cells that were incubated with different concentrations of G-PL for 20 min; n = 3.** (G)** and **(H)** IFN-γ and TNF-α concentrations in the supernatants collected from the co-culture system of **(C)** and **(D)**; n = 5. **(I)** Division of T cells according to the CFSE signal after 48 h tumour-DC-T cell co-culture. The ratio of T cells with low CFSE signals is presented. **(J)** IL-2 concentration in the supernatants at 0 h, 24 h, and 48 h after tumour-DC-T cell co-culture; n = 5.(*: p < 0.05, **: p < 0.01, ***: p < 0.001).

**Figure 3:**
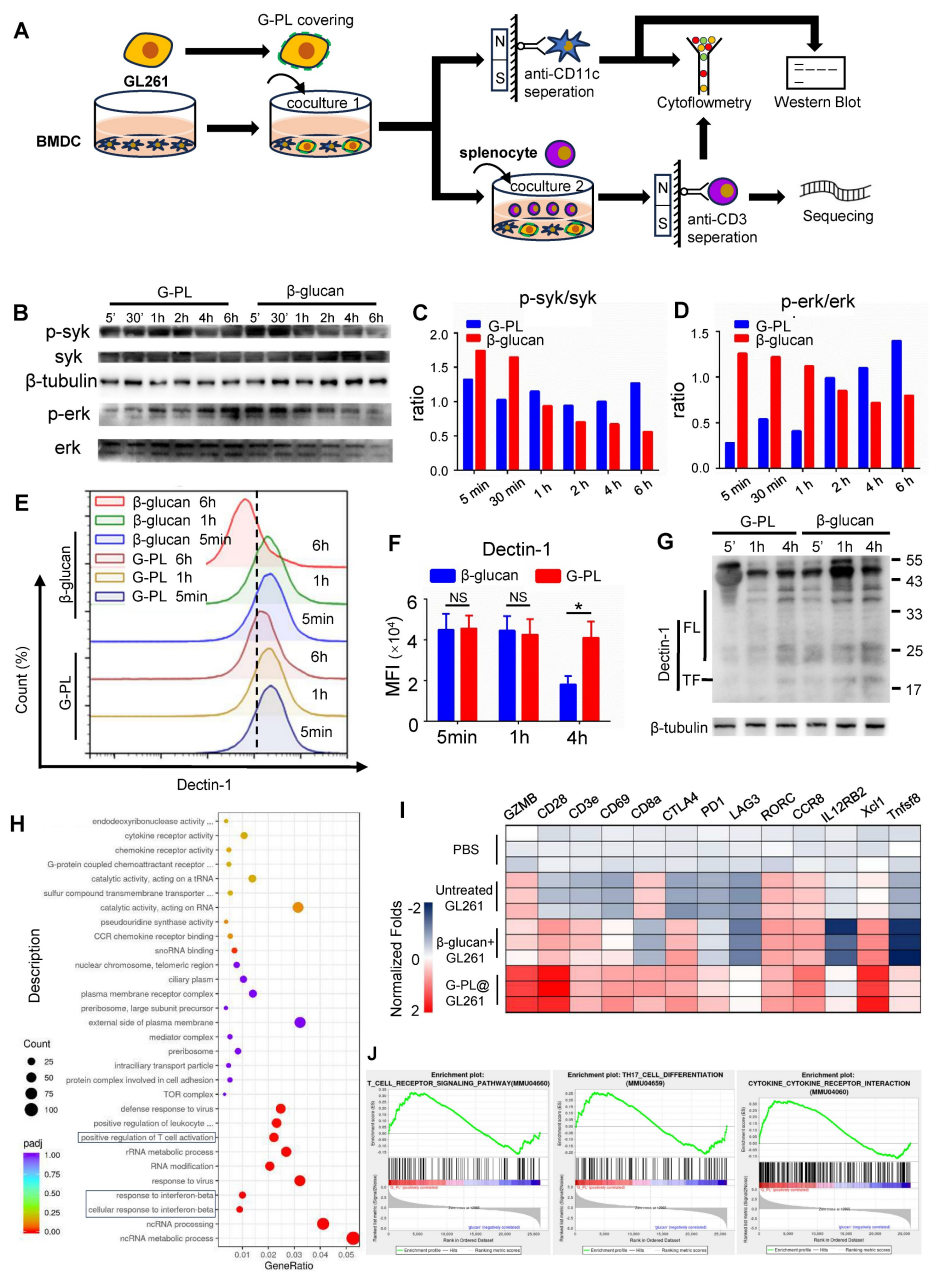
** Membrane-loaded G-PL results in durable activation of the Syk-Erk pathway and the development of Th17 (A)** Schematic representation of the co-culture and analysis process. **(B)** BMDCs were co-cultured with GL261 cells covered with G-PL or soluble β-glucan, and separated by anti-CD11c microbeads for western blot analysis. Representative immunoblots showing the phosphorylation of Syk and Erk in BMDCs that were stimulated at 5 min, 30 min, 1 h, 2 h, 4 h, and 6 h, indicated that the membrane-attached G-PL increased the stability of Dectin-1. The experiment was repeated three times, yielding similar results. **(C) and (D)** Quantification of the phosphorylation of Syk and Erk detected in **(B)**. **(E)** Flow cytometry analysis of the surface expression of Dectin-1, the β-glucan receptor. **(F)** Mean MFI of Dectin-1 expression measured in **(E)**. **(G)** Western blot analysis of the degradation of Dectin-1 during BMDC-tumour co-culture; n = 3. **(H)** GO enrichment analysis of the transcriptome between T cells separated from 72-h Tumour-DC-T cells co-culture with GL261 glioblastoma cells covered with G-PL or soluble β-glucan. **(I)** Representative upregulated genes correlated with T cell activation. **(J)** GSEA plots of indicated signature genes enriched in the BMDCs undergoing co-culture.

We next examined the adjuvant properties of G-PL. To assess DC uptake of tumour antigens, CFSE-labelled GL261 cells were co-cultured with Cytotell Red-labelled immature DCs for 2 h. Confocal microscopy showed that DCs internalised components of G-PL-coated GL261 cells, but not native GL261 cells or GL261 cells with soluble β-glucan (**Figure 2A**). This was also confirmed by flow cytometry, showing that nearly half of the DCs had ingested sufficient GL261 components, including tumour antigens (**Figure 2B**). DC maturation, as assessed by CD80 and CD86 expression, was highest after co-culture with G-PL-coated GL261 for 48 h; whereas native GL261 provided insignificant stimulation, and soluble β-glucan showed positive but lower efficacy (**Figure 2C-D**). G-PL also activated RAW264.7 cells and macrophages (**Figure S2A-B**). ELISA of the supernatants showed that G-PL-coated GL261 significantly induced higher secretion of TNF-α and IFN-β, cytokines typical of mature DCs (**Figure 2G-H**). DC maturation induced by GL261 cells encapsulated with gradient G-PL concentrations required a concentration > 125 μg/mL for significant CD80 and CD86 upregulation (**Figure 2E-F**). Finally, we monitored the activation of T cells induced by co-cultured DCs (**Figure 3A**). The CFSE dilution assay showed the greatest T cell proliferation in the G-PL group (**Figure 2I**), consistent with IL-2 secretion patterns (**Figure 2J**). Moreover, T cells from the G-PL group exhibited significant cytotoxicity against GL261 cells (**Figure S2C**). These results verify the immuno-activating efficacy of G-PL in inducing maturation of APCs and development of tumour-specific T cells.

### Membrane-loaded G-PL results in durable activation of the Syk-Erk pathway and development of Th17

Next, we investigated the mechanism underlying the more effective stimulation of DCs by membrane-bound G-PL than soluble β-glucan. BMDCs and T cells were analysed after co-culture of tumour cells, BMDCs, and splenocytes (**Figure 3A**). WB showed that BMDCs stimulated by membrane-bound G-PL underwent moderate and sustained Syk phosphorylation, leading to progressively increasing Erk phosphorylation. In contrast, soluble β-glucan induced rapid but transient Syk and Erk phosphorylation (**Figure 3B-D**). We hypothesised that this was due to the downregulation of Dectin-1 receptor. Flow cytometry confirmed that Dectin-1 surface expression on the BMDCs decreased significantly after co-culture in the soluble β-glucan group, much more so than in the G-PL group (**Figure 3E-F**). WB also verified increased Dectin-1 degradation in the soluble β-glucan group at 5 min, 1 h, and 6 h post-co-culture compared with the G-PL group (**Figure 3G, S3A**). RNA-seq analysis indicated upregulation of genes related to T cell activation and type I interferon signalling in the G-PL group compared with the soluble β-glucan group (**Figure 3H, I**). Gene Set Enrichment Analysis (GSEA) suggested that T cells in the G-PL group received stronger T cell receptor (TCR) activation signals and underwent greater Th17 differentiation and cytokine stimulation (**Figure 3J**). Flow cytometry findings supported this observation, showing increased IL-17 and IFN-γ expression in CD4^+^ T cells from the G-PL group (**Figure S3B-C**). These results indicate that G-PL stimulation reduces Dectin-1 degradation, leading to enhanced T cell activation and Th17 differentiation.

### ICC@G-PL vaccination prompts the development of anti-tumour adaptive immunity

We constructed a whole cancer cell vaccine (WCCV) by coating irradiated cancer cells with G-PL (denoted by ICC@G-PL) and evaluated its efficacy *in vivo*. First, subcutaneous injection of G-PL alone (two 500-μg doses) in GL261 xenograft mice did not significantly improve survival (**Figure S4A**).

**Figure 4:**
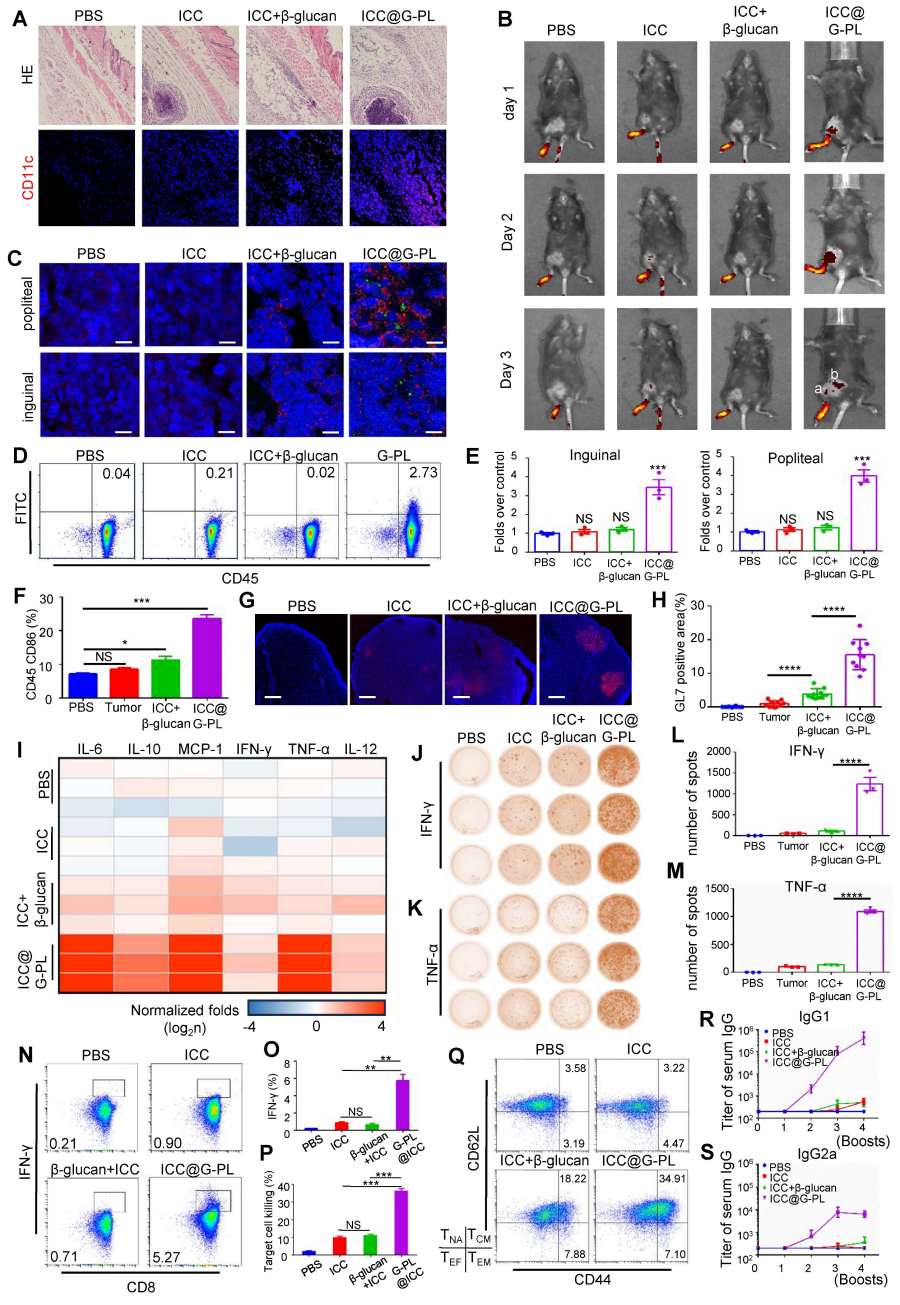
** ICC@G-PL vaccination prompts the development of anti-tumour adaptive immunity (A)** H&E and immunofluorescence staining of the injection site of ICC@G-PL, demonstrating that significant inflammation was induced by the ICC@G-PL with DC infiltration at the injection site. Scale bars, 500 μm for H&E and 100 μm for immunofluorescence images. The specimens were collected 48 h after injection from the back of the mouse. The preparation of ICC, ICC+β-glucan, and ICC@G-PL groups was the same as that of untreated GL261, GL261+β-glucan, and GL261@G-PL, except that the primary GL261 cells were irradiated at a dose of 6 Gy before use. **(B)** *In vivo* imaging of the uptake of DID-labelled GL261 antigen, showing increased antigen uptake in draining lymph nodes induced by G-PL. a: popliteal; b: inguinal. **(C)** Confocal monitoring of the CFSE-labelled GL261 antigen (green) uptake in popliteal and inguinal lymph nodes 48 h after footpad injection. The DCs in the lymph nodes were labelled with anti-CD11c (red). Scale bar, 40 μm.** (D)** Flow cytometry analysis of the proportion of DCs in the popliteal nodes taking in the CFSE-labelled antigen. **(E)** Enlarged volume of the popliteal and inguinal lymph nodes compared with that of the blank control, n = 3. **(F)** Flow cytometry analysis of CD86 expressed by CD45 APC cells in popliteal lymph nodes, n = 3. **(G)** Confocal microscopy of popliteal lymph nodes with antibodies against GL-7 (red), indicating the activation of B cells in the draining lymph nodes. DAPI was used for nuclear counterstaining. One representative image per group is shown. Scale bar, 1 mm. **(H)** Quantification of the proportion of GL-7-positive area. **(I)** Inflammatory cytokines in the serum of vaccinated mice were detected using Cytometric Bead Array 72 h post-vaccination (n = 3 mice/group). The results were normalised to the PBS control group. **(J) and (K)** Elispots analysis of the IFN-γ- and TNF-α-secreting splenocytes, showing that the use of G-PL significantly increased the development of tumour-specific T cells. **(L) and (M)** Mean±SD of the IFN-γ and TNF-α spots per well of each group, n = 3. **(N) and (O)** Flow cytometry analysis of the IFN-γ-secreting CD8^+^T cells among splenocytes removed from the vaccinated mice after a 48-h co-culture of splenocytes and GL261 cells, n = 3. **(P)** Apoptosis of the GL261 cells co-cultured with splenocytes from the vaccinated mice 48 h post co-culture. Splenocytes:GL261=50:1, n = 3. **(Q)** Flow cytometry analysis of the CD44^+^/CD62L^+^ memory T cells (T_EF_). The experiment was performed three times, yielding similar results. T cell subtypes: CD44^lo^/CD62L^lo^-effector T cells, CD44^lo^/CD62L^hi^-naive T cells (T_NA_), CD44^hi^/CD62L^lo^-effector memory T cells (T_EM_), CD44^hi^/CD62L^hi^-central memory T cells (T_CM_). **(R) and (S)** ELISA of GL261 lysate-specific IgG1 and IgG2a antibody titres. Data represent the mean ± SEM, n = 3.

Although G-PL alone induced lymph node DC maturation (**Figure S4B**), it failed to generate tumour-specific T cells upon subsequent GL261-splenocyte co-culture (**Figure S4C**), indicating the necessity of the co-delivered antigen. We determined that irradiation (6 Gy) effectively induced GL261 apoptosis (**Figure S4D-E**) and abrogated tumorigenicity *in vivo* (**Figure S4F**), thus standardising ICC@G-PL preparation for glioblastoma models with this dose. Subcutaneous inoculation of ICC@G-PL induced significant inflammation and DC recruitment at the injection site within 3 days (**Figure 4A, S4G**). Footpad injection of DID-labelled ICC@G-PL showed antigen accumulation in the popliteal lymph node by day 2, extending to the inguinal node by day 3 (**Figure 4B**). Immunofluorescence confirmed DC migration and CFSE-labelled tumour antigen accumulation in these nodes at 48 h (**Figure 4C**), which was quantified by flow cytometry (**Figure 4D**). The draining lymph nodes in the G-PL groups were significantly enlarged by day 3 (**Figure 4E, S4H**) and showed increased CD86 expression on APCs (**Figure 4F**). By day 10, the lymph nodes exhibited GL-7+ germinal centres, indicating B and T cell activation (**Figure 4G-H**). The cytometric bead array 72 h post-vaccination showed elevated serum levels of IL-6, IL-10, MCP-1, and TNF-α (**Figure 4I, S5**). After two vaccinations, splenocytes from ICC@G-PL-vaccinated mice co-cultured with tumour cells produced more IFN-γ and TNF-α spots (ELISPOT) and secreted higher levels of these cytokines (ELISA), indicating tumour-specific T cell development (**Figure 4J-M**). Flow cytometry confirmed increased tumour-specific CD8^+^ T cells (**Figure 4N-O**) and enhanced splenocyte cytotoxicity against GL261 (**Figure 4P**). Mice receiving four booster vaccinations every two weeks developed higher serum IgG, IgG1, and IgG2a titres against GL261 lysates (**Figure 4R-S, S6B-C**), and an increased proportion of memory T cells (CD44+/CD62L+) (**Figure 4Q, S6A**). Repeated ICC@G-PL vaccination caused no significant organ damage compared with controls (**Figure S7A-B**).

### Prophylactic and therapeutic ICC@G-PL vaccination induces tumour regression in a murine glioblastoma model

**Figure 5:**
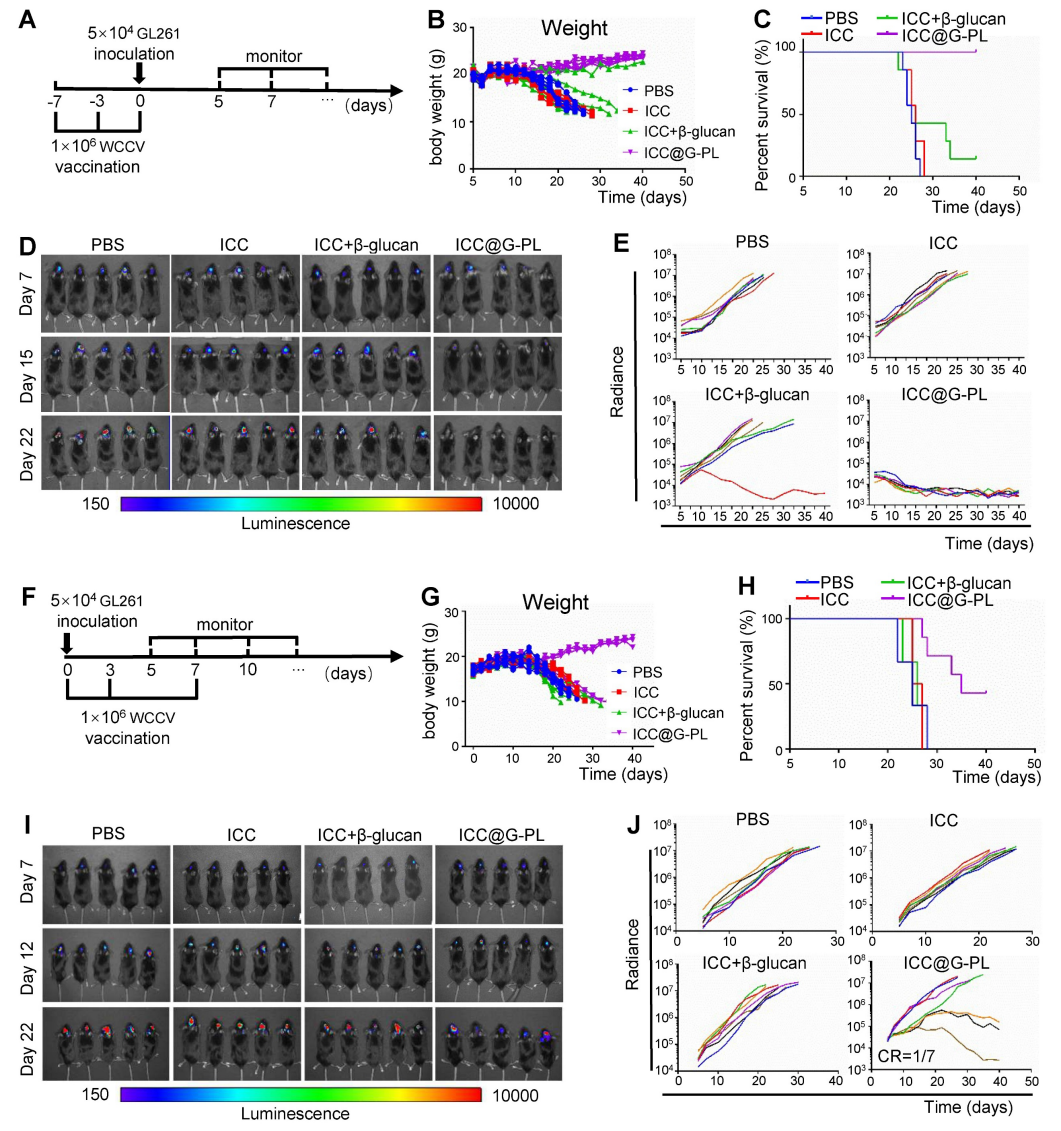
** Prophylactic and therapeutic ICC@G-PL vaccination induce tumour regression in a murine glioblastoma model (A)** Schematic diagram of the anti-tumour efficacy of preventive vaccination in GL261 xenograft model. In the preventive vaccination model, the mice were vaccinated 7 days and 3 days before intracranial inoculation of GL261 glioblastoma cells. The growth of the tumour was monitored by IVIS three times each week, starting on day 5. **(B)** Change in body weight of the mice in each group of the preventive vaccination model. **(C)** Survival of the mice in each group of the preventive vaccination model (n = 7) **(D)** *In vivo* bioluminescence images of control and vaccinated mice bearing GL261-Lucf tumours of the preventive vaccination model. **(E)** Individual tumour growth curves for each group in **(D)** according to the measured radiance. **(F)** Schematic diagram of the anti-tumour efficacy of therapeutic vaccination in the GL261 xenograft model. In the therapeutic vaccination model, the mice were vaccinated on the same day as GL261 inoculation and again on days 3 and 7 after GL261 inoculation. The growth of the tumour was monitored by IVIS three times each week, starting on day 5. **(G)** Change in body weight of the mice in each group of the therapeutic vaccination model. **(H)** Survival of the mice in each group of the therapeutic vaccination model. **(I)** *In vivo* bioluminescence images of control and vaccinated mice bearing GL261-Lucf tumours of the therapeutic vaccination model. **(J)** Individual tumour growth curves for each group in **(I)** according to the measured radiance.

After confirming the ability of ICC@G-PL to activate dendritic cells (DCs) and T cells *in vivo*, we proceeded to evaluate its therapeutic efficacy in xenograft models. Initially, we investigated the prophylactic potential of ICC@G-PL vaccination (**Figure 5A**), in which mice received three consecutive ICC@G-PL vaccinations prior to GL261 tumour inoculation. The results demonstrated that the mice in the ICC@G-PL group did not undergo body weight loss, and all of them survived the GL261 challenge (**Figure 5B-C**). Notably, soluble β-glucan also provided some benefit to a subset of xenografts. Tumour progression was monitored three times a week using IVIS, revealing that both the WCCV with soluble β-glucan and ICC@G-PL-vaccinated mice exhibited attenuated tumour progression, as indicated by the reduced radiance. While all seven mice in the ICC@G-PL group achieved complete tumour regression, only one of seven mice in the WCCV with soluble β-glucan group showed complete regression (**Figure 5D-E**). Next, we evaluated the therapeutic efficacy of ICC@G-PL in a therapeutic vaccine model, wherein xenograft mice were vaccinated on days 0, 3, and 7 after GL261 inoculation (**Figure 5F**). The results indicated that three out of seven mice survived following therapeutic ICC@G-PL vaccination (**Figure 5G-H**). Tumour growth was monitored via IVIS, and ICC@G-PL-treated mice exhibited a significant reduction in tumour burden (**Figure 5I-J**). Notably, one out of seven mice in the ICC@G-PL group achieved complete tumour regression within 40 days of monitoring. To assess long-term immunity, we rechallenged this mouse with an intracranial inoculation of 1 × 10^5^ GL261 cells and observed that it remained resistant to tumour regrowth (**Figure S8**).

### Therapeutic ICC@G-PL vaccination leads to increased accumulation of immune cells in the cancer microenvironment

**Figure 6:**
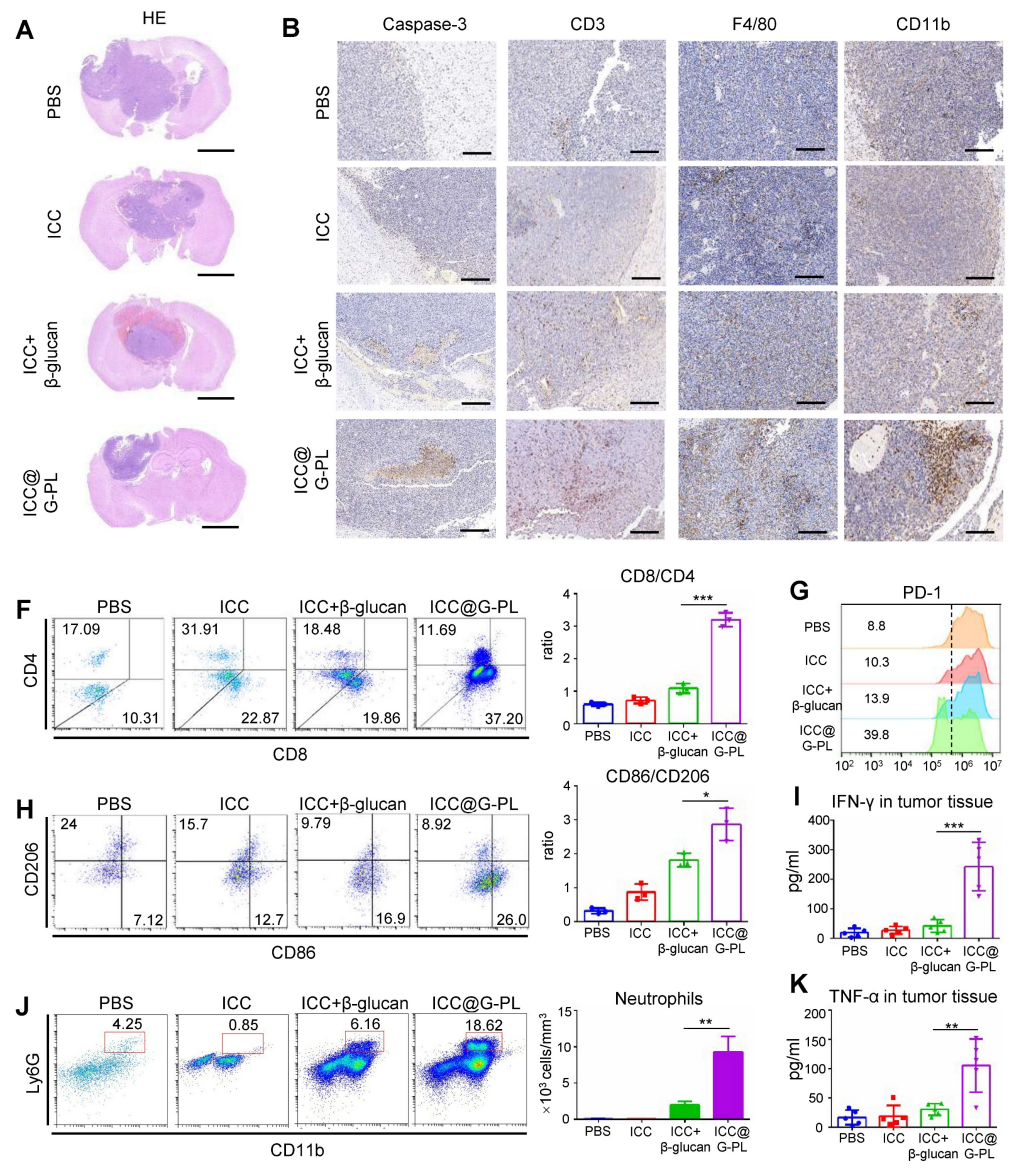
** Therapeutic ICC@G-PL vaccination leads to increased accumulation of immune cells in the cancer microenvironment. (A)** Representative photographs of H&E staining of the xenograft sections removed from mice of the therapeutic vaccination model. **(B)** Immunohistochemistry assay was conducted for monitoring tumour apoptosis and infiltration of T cells, macrophages, and neutrophils according to cleaved Caspase-3, CD3, F4/80, and CD11b expression levels. Scale bar, 2 mm. **(C)** Flow cytometry analysis of the tumour-infiltrated CD4^+^ and CD8^+^ T cells. The CD8/CD4 ratio was calculated and presented as mean±SD (n = 3). **(D)** Flow cytometry analysis of the tumour-infiltrated M1 (CD86) and M2 (CD 206) macrophages. The M1/M2 ratio was calculated and presented as mean±SD (n = 3). **(E)** Flow cytometry analysis of the tumour-infiltrated neutrophils (CD11b^+^/Ly6G^+^); the number of neutrophils in 1 mm^3^ of tumour tissue was calculated and presented as mean±SD (n = 3). **(F)** PD-1 expression in the tumour-infiltrated T cells. The percentages of pooled PD-1^lo^ T cells are shown. The experiment was repeated thrice, yielding similar results. **(G)** and **(H)** IFN-γ and TNF-α concentrations in the xenograft tumour were measured by ELISA (n = 5). ***: p < 0.001.

To further explore the mechanisms underlying ICC@G-PL-mediated tumour regression, we conducted a parallel experiment in which the mice were sacrificed on day 20 for the analysis of immune cell infiltration within the xenograft tumours. Haematoxylin and eosin (H&E) staining revealed that ICC@G-PL vaccination significantly reduced the tumour volume (**Figure 6A**). Immunohistochemical staining for cleaved Caspase-3 indicated tumour necrosis in all mice that received WCCV vaccination, a feature not observed in control mice treated with PBS (**Figure 6B**).

Furthermore, we observed that WCCV-treated mice exhibited enhanced T cell infiltration (CD3), with ICC@G-PL showing the greatest effect. Interestingly, the use of β-glucan as an adjuvant promoted the accumulation of myeloid cells (CD11b), particularly in the G-PL group. Tumour-infiltrating macrophages (F4/80) were observed across all groups, although their distribution did not differ significantly among the treatment conditions. To further investigate the immune cell subtypes, flow cytometry was used to analyse the infiltrating immune populations (**Figure S9A**). ICC@G-PL vaccination significantly increased the CD8^+^/CD4^+^ T cell ratio (**Figure 6C**) and alleviated T cell exhaustion, as indicated by lower levels of PD-1 expression (**Figure 6F**). Although WCCV vaccination did not increase the number of macrophages as shown by immunohistochemistry, it induced an M1-skewed macrophage phenotype, particularly in the ICC@G-PL-treated group (**Figure 6D**). Furthermore, ICC@G-PL vaccination led to a significant increase in the percentage of neutrophils (CD11b^+^/Ly6G^+^) in the tumour microenvironment (**Figure 6E**), with a predominant Ly6G^+^/Ly6C^-^ phenotype (**Figure S9A**). Importantly, tumour infiltration by neutrophils was markedly reduced following IL-17 blockade in a parallel study (**Figure S9B**). Consistent with these cellular changes, elevated levels of IFN-γ and TNF-α were detected in the tumour tissues of ICC@G-PL-treated mice (**Figure 6G-H**).

### Postoperative use of ICC@G-PL inhibits tumour progression in multiple murine models

**Figure 7:**
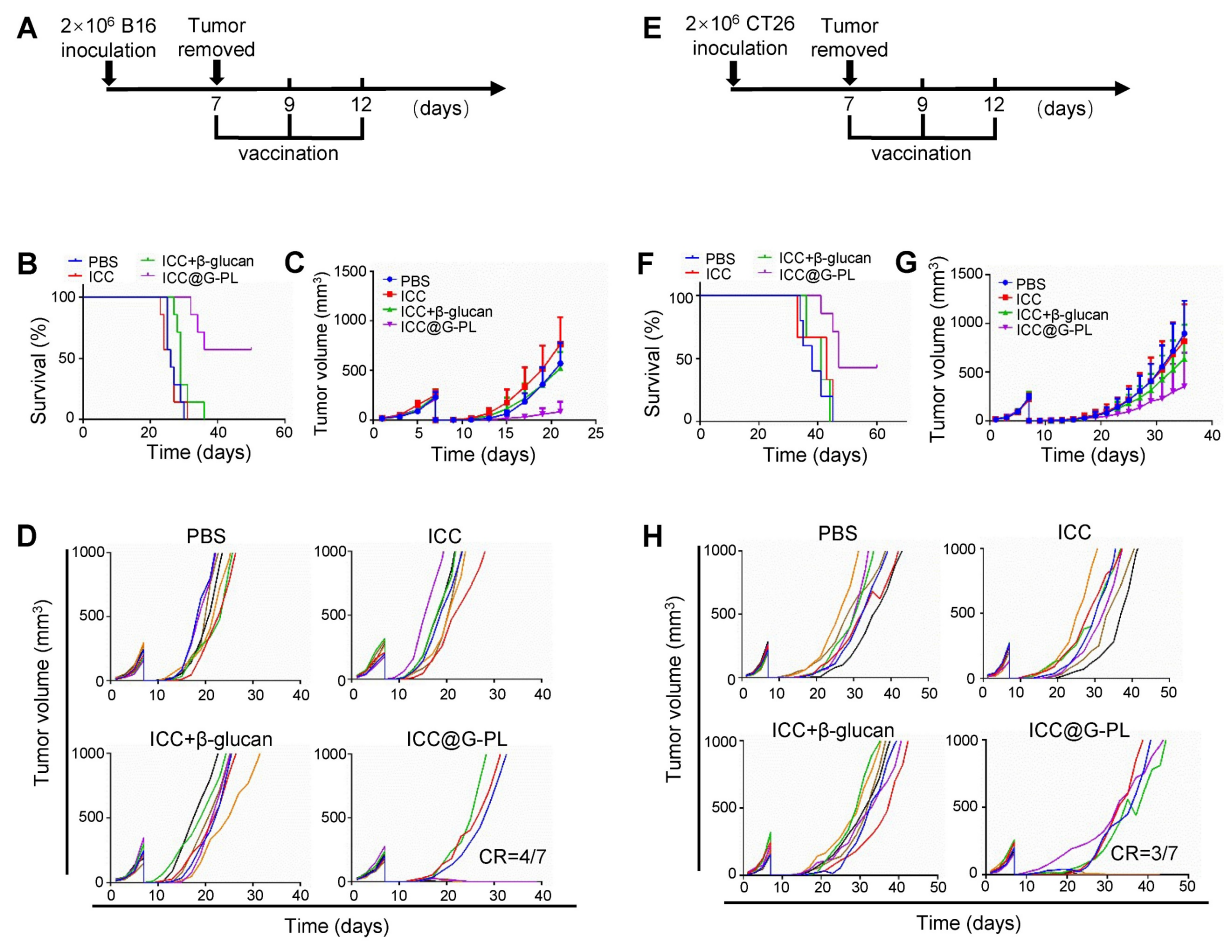
** Use of ICC@G-PL post-debulking surgery inhibits tumour progression in multiple murine models.** The therapeutic efficacy of ICC@G-PL was further verified in murine melanoma B16 and murine colon cancer CT26 subcutaneous models. In both the models, the xenograft tumours were removed on day 7 and then developed into WCCVs for vaccination. **(A)** Schematic diagram of the B16 model. **(B)** Survival curve of the mice bearing B16 xenografts receiving each treatment. **(C)** B16 tumour volume in the mice receiving each treatment was recorded every other day. **(D)** Individual B16 tumour growth curve of each group of mice in **(C)**. **(E)** Schematic diagram of the CT26 model. **(F)** Survival curve of the mice bearing CT26 xenografts receiving each treatment. **(G)** CT26 tumour volume in the mice receiving each treatment was recorded every other day. **(H)** Individual CT26 tumour growth curve of each group of mice in **(G)**. The data are presented as means ± SD.

To investigate the potential of ICC@G-PL as a post-surgical immunotherapy, we used subcutaneous tumour models wherein the tumours were surgically resected on day 7 and treated with ICC@G-PL following irradiation (7.5 Gy for B16 and 12 Gy for CT26) and G-PL coating. We first assessed the therapeutic efficacy in the B16 melanoma model (**Figure 7A**). The mice received three doses of ICC@G-PL on days 7, 9, and 12 post-surgery. ICC@G-PL treatment significantly improved survival and inhibited tumour growth (**Figure 7B-C**). Four out of seven mice in the ICC@G-PL group remained free of tumour recurrence, whereas the other three mice showed slower tumour growth than mice in the control groups (**Figure 7D**).

We then evaluated the postoperative efficacy of ICC@G-PL in the CT26 colon cancer model (**Figure 7E**). Similar to the B16 model, subcutaneous inoculation of 2×10^6^ CT26 cells was followed by tumour resection and ICC@G-PL treatment. As expected, ICC@G-PL vaccination significantly suppressed tumour recurrence and growth, leading to improved survival in the treated mice (**Figure 7F-G**).

## Discussion

Vaccines have shown great potential in fighting against infectious diseases, but have failed to induce lasting anti-tumour immune responses. Further, they only extend patient survival by months when used in cancer treatment due to the weak immunogenicity of tumour antigens and lack of universally-expressed TSAs as a result of tumour heterogeneity 14, 15. Previous studies have shown that the chimeric vaccination system combining microbial components with tumour antigens can successfully broaden the immune responses to include cancer epitopes 16. However, most of the existing co-delivery systems are based on peptides or tumour lysates 17, and such a design for WCCVs is still absent. We constructed the first WCCV and β-glucan co-delivery system based on novel adjuvant G-PL, and explored its efficacy *in vitro* and *in vivo*.

Yeast-derived β-glucan is the major component of the yeast cell wall and plays an important role in the development of adaptive anti-fungal immunity. As an immunomodulator, β-glucan exhibits a safe profile and is approved to be a food additive by the US Food and Drug Administration 18. To construct an antigen-glucan co-delivery system, we designed a novel compound by conjugating the yeast-derived β-glucan with poly-lysine, which is a positively-charged carrier peptide widely used in cellular drug delivery in previous studies 19. This compound can adhere to the cell membrane via the positive-charged poly-lysine tail. Although it is reported that rapid cellular intake follows the membrane loading of poly-lysine, this process can be hindered by using poly-D-lysine and coupling it with cargos 20, 21. This is in accordance with our results (**Figure 1**) indicating that G-PL-FITC mainly aggregated on the membrane of the coated cell with mild intake and cytotoxicity. We further demonstrated the immuno-activating efficacy of G-PL *in vitro* and *in vivo* including multiple tumour models.

We also explored the mechanisms underlying the therapeutic efficacy. The study of the receptor of β-glucan, Dectin-1, is of particular interest since only the particulate form of its ligands induces efficient receptor clustering and activation 22. Previous research has tried to exploit the immunomodulatory capacity of insoluble β-glucan particles, with peptides, RNA, or tumour lysate antigens inserted into them 23. However, a recent study showed that the weak immunogenicity of soluble C-type Lectin Receptor ligands with small-sized particles can be reversed by modulating their physical properties such as by adsorbing the CLR ligands onto alum 24. Based on this discovery, we supposed that cell-coated G-PL can also lead to intact immune activation. We found that our co-delivery system significantly induced antigen uptake, maturation, cytokine release of DC, and development of tumour-specific T cells. Detailed exploration also revealed that, compared with soluble β-glucan, the use of G-PL led to a mild and delayed but long-lasting activation of the Dectin1-Syk-Erk pathway, and preserved the membrane Dectin-1 stability from upregulation and degradation, all of which are similar to the previously reported results of insoluble β-glucan particles 25. It is worth noting that, to avoid the immunomodulatory effect exerted by irradiated cells on DC, we coated wild murine GL261 glioblastoma cell lines with G-PL adjuvant during these *in vitro* experiments but not the 6-Gy-irradiated GL261 cells used in the construction of ICC@G-PL for *in vivo* experiments. Moreover, the co-culture of activated DC cells and T cells led to the development of a group of Th17 cells. Th17 induced by DCs is reported to have greater therapeutic efficacy 26. However, this cell type is also associated with many inflammatory and autoimmune disorders. Non-pathogenic Th17 is recognised as IFN-γ negative and plays an important role in anti-fungal immunity 27. Our results showed that the Th17 induced by G-PL was non-pathogenic and may provide additional benefits beyond those offered by traditional cancer vaccines.

In the current study, we have demonstrated the technical feasibility and functional capacity of G-PL to induce both innate and tumour-specific adaptive immune responses. More importantly, our findings provide additional advantages for clinical translation. First, G-PL was verified to be an applicable strategy to construct a WCCV-adjuvant system. Besides β-glucan, the poly-lysine modification can be used for attachment of other types of immunomodulators to the cell membrane, including mannan, GM-CSF, and even immunoglobulins. Second, this strategy is based on live-attenuated cancer cells, in which the cancer cells can be further modified by gene editing. Third, since surgery is still the primary treatment for most types of tumours, the autologous cancer cells are available. Finally, as our strategy can be dispensed without gene editing or sequencing and the adjuvant is readily produced, the patient can receive the vaccination at an early-stage post-surgery. We also observed improved survival associated with prophylactic vaccination compared with therapeutic vaccination, which may be attributed to lower tumour burden and construction of adaptive anti-tumour immunity.

One limitation of our study is that we have not explored the post-treated tumour microenvironment, especially the tumour-infiltrated neutrophils in the ICC@G-PL-treated group, in detail. Previous reports suggest that neutrophils in the tumour can be divided into different subtypes leading to either pro-tumour or anti-tumour activity such as angiogenesis, metastasis, immunosuppression, or direct tumour killing. In our study, we observed significantly increased neutrophil infiltration in the post-treated xenograft models. However, the exact subtype and function of these neutrophils remain unclear, which may be of special importance for the exploration of further combined therapy. Moreover, we have observed Th17 development during the *in vitro* co-culture of murine splenocytes and G-PL-activated DCs. Although it is reported that Th17 can recruit neutrophils to induce tissue inflammation, whether or not the tumour-infiltrating neutrophils found in the post-treated mice in our *ex vivo* study were recruited by tumour-specific Th17 remains to be explored in future studies.

## Conclusion

This study successfully developed a novel conjugate adjuvant G-PL by coupling yeast-derived modified β-glucan with poly-D-lysine, and constructed an autologous WCCV (ICC@G-PL) by coating irradiated tumour cells with G-PL. This strategy addresses two critical limitations of conventional WCCVs—weak immunogenicity and cumbersome preparation procedures—providing a universal and clinically translatable platform for personalised cancer immunotherapy.

First, we verified the core properties of G-PL through a series of *in vitro* experiments. G-PL exhibited a positive zeta potential (28±0.1 mV) and diameter of 134.6±55.8 nm, enabling rapid and stable electrostatic binding to the negatively charged membrane of tumour cells (e.g., GL261, B16, CT26). Notably, G-PL (≤500 μg/mL) showed no significant cytotoxicity or haemolytic activity towards RBCs, ensuring its biocompatibility for *in vivo* applications.

Second, mechanistically, G-PL significantly enhanced anti-tumour immune responses by regulating the Dectin-1-Syk-Erk signalling pathway. Compared with soluble β-glucan (which induces rapid but transient pathway activation and Dectin-1 degradation), membrane-bound G-PL triggered moderate but durable phosphorylation of Syk and Erk in dendritic cells (DCs), reduced Dectin-1 degradation, and promoted DC maturation (upregulated CD80/CD86) and non-pathogenic Th17 cell differentiation. This unique mechanism not only strengthened DC-mediated antigen cross-presentation but also avoided the risk of autoimmune disorders associated with pathogenic Th17 cells.

*In vivo* studies further confirmed the prophylactic and therapeutic efficacy of ICC@G-PL. In the murine glioblastoma (GL261) prophylactic model, ICC@G-PL vaccination achieved 100% survival (7/7 mice) and complete tumour regression, outperforming the soluble β-glucan group (only 1/7 mice with complete regression). In the therapeutic model, ICC@G-PL prolonged survival (3/7 mice survived) and reduced tumour burden, accompanied by reprogramming of the tumour microenvironment: increased intratumoral CD8⁺/CD4⁺ T cell ratio, M1-type macrophage polarisation (3.8-fold higher than control), and neutrophil infiltration. Moreover, ICC@G-PL induced robust adaptive immunity, including elevated serum levels of anti-tumour cytokines (IL-12, TNF-α, IFN-γ), increased tumour-specific CD8⁺ T cells, and enhanced memory T cell formation (CD44⁺/CD62L⁺), which contributed to long-term anti-tumour protection (e.g., resistance to GL261 rechallenge in cured mice).

Finally, ICC@G-PL demonstrated broad applicability and clinical potential in multiple tumour models. In post-debulking surgery models of melanoma (B16) and colon cancer (CT26), ICC@G-PL significantly inhibited tumour recurrence (4/7 mice free of B16 recurrence) and improved overall survival, without causing obvious organ damage (e.g., heart, liver, spleen) in long-term vaccinated mice. This advantage is particularly critical for clinical translation, as ICC@G-PL can be prepared using autologous tumour tissues obtained from surgical resection—without the need for gene editing, neoantigen sequencing, or prolonged *in vitro* cell culture—enabling early postoperative vaccination for patients.

In summary, our findings confirm that G-PL-based ICC@G-PL is a safe, efficient, and easy-to-prepare WCCV. It enhances tumour-specific immunity, reprograms the immunosuppressive tumour microenvironment, and exhibits broad anti-tumour efficacy across multiple solid tumours. Future studies will focus on clarifying the subtype and functional role of tumour-infiltrating neutrophils, as well as optimising the formulation to further improve its efficacy in combination with immune checkpoint inhibitors (e.g., anti-PD-1), thereby laying a solid foundation for subsequent clinical trials.

## Supplementary Material

Supplementary figures.
