# Supplementary material for: A novel whole cancer cell vaccine based on modified β-glucan elicits robust anti-tumor immunity
Source: Theranostics. 2026 Jan 1;16(6):2936–51. doi: 10.7150/thno.121911 (PMC12775824; doi:10.7150/thno.121911)
Supplement: Supplementary file 1 — Supplementary figures. [file thnov16p2936s1.pdf]

Supplementary Figure Legends

Supplementary Figure 1. Size distribution and Zeta potential of β-glucan and haemolytic tests for different concentrations of G-PL. **(A)** Process of G-PL synthesis. **(B)** Characteristic peaks corresponding to β-glucan and poly-L-lysine. **(C)** n = 3, biologically independent replicates. Data are presented as mean ± SD. **(D)** A series of concentrations of G-PL was added to the erythrocytes. Haemolysis was assessed 1 h later. Haemolysis was observed when the G-PL concentration was higher than 1 mg/mL.

Supplementary Figure 2. **(A)** and **(B)** Activation of RAW264.7 by G-PL. G-PL-coated GL261 cells were co-cultured with RAW264.7 for 48 h, and the CD80 and CD86 expressed on macrophages were analysed by flow cytometry. **(C)** Co-culture of the splenocytes, DCs, and GL261 cells. A total of 1×10^5 GL261 cells or PBS was co-cultured with 1×10^5 DCs; 48 h later, 1×10^6 splenocytes were added for further co-culture to monitor the development and cytotoxicity of GL261-specific T cells according to the apoptosis of GL261 and proliferation of T cells. In the PBS group, GL261 cells were not added to the co-culture system, and PBS solution was used instead. In the glucan group, 500 μg/mL β-glucan was added to the co-culture supernatant. In the G-PL group, the GL261 cells were pretreated with 500 μg/mL G-PL solution and then added to the co-culture system. Representatives of tumour cells that were alive are marked with arrows. Scale bar, 200 μm.

Supplementary Figure 3. **(A)** The ratio of terminal fragment versus full length of Dectin-1 and the T cell subtypes after tumour-DC – T cells co-culture. DCs co-cultured with GL261 cells were collected at different time points after co-culture. GL261 cells were pretreated with 500 μg/mL G-PL solution before co-culture in G-PL group. In the glucan group, the GL261 cells were not pre-coated with G-PL, but 500 μg/mL β-glucan was added to the supernatant of the co-culture medium instead. (B) T cells were separated from the Tumour – DC – splenocytes co-culture system by using anti-CD3 magnetic beads. For the T cell subtype analysis, IFN-γ and IL-17 expressed by $CD4^+$ T cells were used for Th1 and Th17 detection during flow cytometry analysis. **(C)** The average of three replicates was calculated. **(D)** and **(E)** WT and Dectin-1 KO mice were independently subcutaneously inoculated with 1×10^5 ICC@G-PL, and the Th17 ratio in splenocytes was analysed 7 days later.

Supplementary Figure 4. **(A)** Xenograft mice were inoculated with 500 μg G-PL or PBS (control) twice on days 0 and 7 following GL261 inoculation. No survival benefits were found. **(B)** Inguinal lymph nodes were extracted 48 h later to measure the DC maturation level based on CD86 expression. **(C)** Splenocytes were extracted on day 20 after inoculation and co-cultured with GL261 at a ratio of 10:1 for 3 days; the expression of IFN-γ was further measured. **(D)**, **(E)**, and **(F)** Apoptosis of GL261 cells induced by gradient irradiation. GL261 cells were cultured and irradiated by a medical accelerator. Cell apoptosis was analysed to find the appropriate dose of

irradiation for ICC@G-PL construction by using Western Blot detection of the apoptosis marker, cleaved Caspase-3, and recording the growth of GL261 cells after gradient radiation exposure *in vitro* and *in vivo* based on cell counting and IVIS radiance, respectively. **(G)** and **(H)** Local inflammation induced by ICC@G-PL during footpad injection. ICC@G-PL cells (1×10^6 in 50 μL of PBS) were inoculated into the footpad of mice, and the injection sites and draining lymph nodes were monitored 48 h later. Significant redness and swelling were observed at the injection site (footpad) after injection of ICC@G-PL, compared with the control group. Enlarged draining lymph nodes were observed. Left to right: PBS, ICC, ICC+β-glucan, ICC@G-PL.

Supplementary Figure 5. Example of the CBA analysis. Serum samples from the different groups of mice were diluted at a ratio of 1:100 and then analysed by CBA. The bead-conjugated antibodies of IL-6, IL-10, MCP-1, IFN-γ, TNF-α, and IL-12 were labelled with PE fluorochrome at different intensities.

Supplementary Figure 6. Humoral immunity induced by ICC@G-PL vaccination. Serum samples from different groups of C57BL/6 mice were collected after an 8-week-long consecutive vaccination, and the titres of tumour-specific IgG1 and IgG2a were measured by ELISA. The endpoint titres were determined as the titre range at which the absorbance was twice the absorbance of the blank. *: $p < 0.05$, **: $p < 0.01$, ***: $p < 0.0001$. Data are represented as mean ± SEM. Statistical significance was calculated by one-way ANOVA with Tukey's post-hoc test for multiple comparison correction. **(A)** Illustration of the experiment design. **(B)** Titration of IgG1 by serial dilution. **(C)** Titration of IgG2a by serial dilution.

Supplementary Figure 7. Major organ damage screening. **(A)** H&E staining of the heart, kidney, spleen, lung, and liver. The organs of the vaccinated mice were collected for H&E staining 10 weeks after the first vaccination. No significant organ damage was observed after the ICC@G-PL vaccination. Scale bar, 200 μm. **(B)** Serum levels of alanine aminotransferase (ALT), aspartate aminotransferase (AST), triglycerides, total cholesterol, uric acid, blood urea nitrogen (BUN), glucose, and amylase were measured. n = 3 for each group.

Supplementary Figure 8. Rechallenge of the treated mouse with GL261 cells. The mouse that underwent complete tumour relief in the therapeutic vaccine model was rechallenged by intracranial inoculation of 1×10^4 GL261 cells. IVIS was conducted every 48 h starting on day 7, and the radiance was recorded. Control group, n = 3.

Supplementary Figure 9. **(A)** Flow cytometry gate strategy for tumour microenvironment analysis. The tumour specimens of the xenograft mice were collected and dissociated into single-cell suspensions. The live immune cells in the tumour were recognised by FVS620$^-$ and CD45$^+$, and the cell subtypes such as T cells, macrophages, and neutrophils were further differentiated by characterised markers. **(B)**

and **(C)** After therapeutic inoculation with ICC@G-PL, the GBM xenograft mice were intraperitoneally injected with 5 mg/kg anti-IL-17A every other day for 2 weeks, starting from the day of GL261 inoculation. For $CD4^+$ and $CD8^+$ T cell depletion, the mice were intraperitoneally injected with four doses of 200 μg per mouse, twice a week. The infiltration of neutrophils and T cells was measured using immunofluorescence on day 20. The survival of the mice was observed in a parallel study.

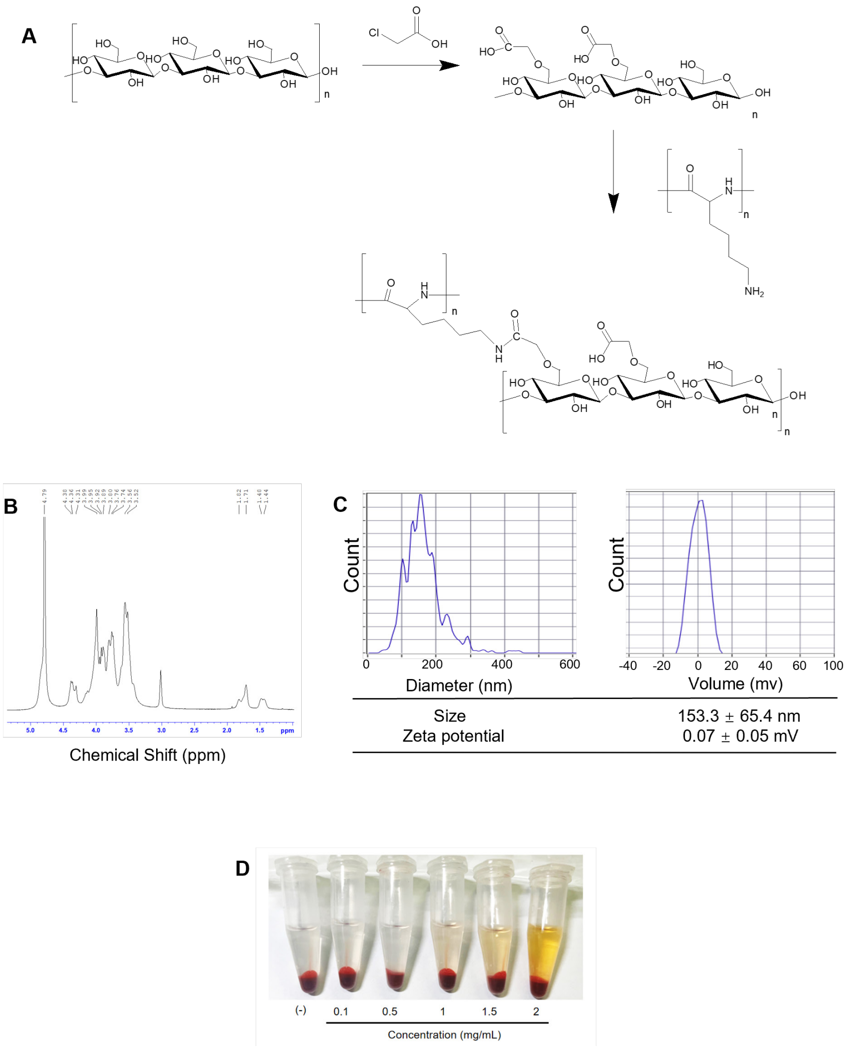
A
B
C
D
Chemical Shift (ppm)
Count
Diameter (nm)
Count
Volume (mv)
Size 153.3 ± 65.4 nm
Zeta potential 0.07 ± 0.05 mV
(-) 0.1 0.5 1 1.5 2
Concentration (mg/mL)

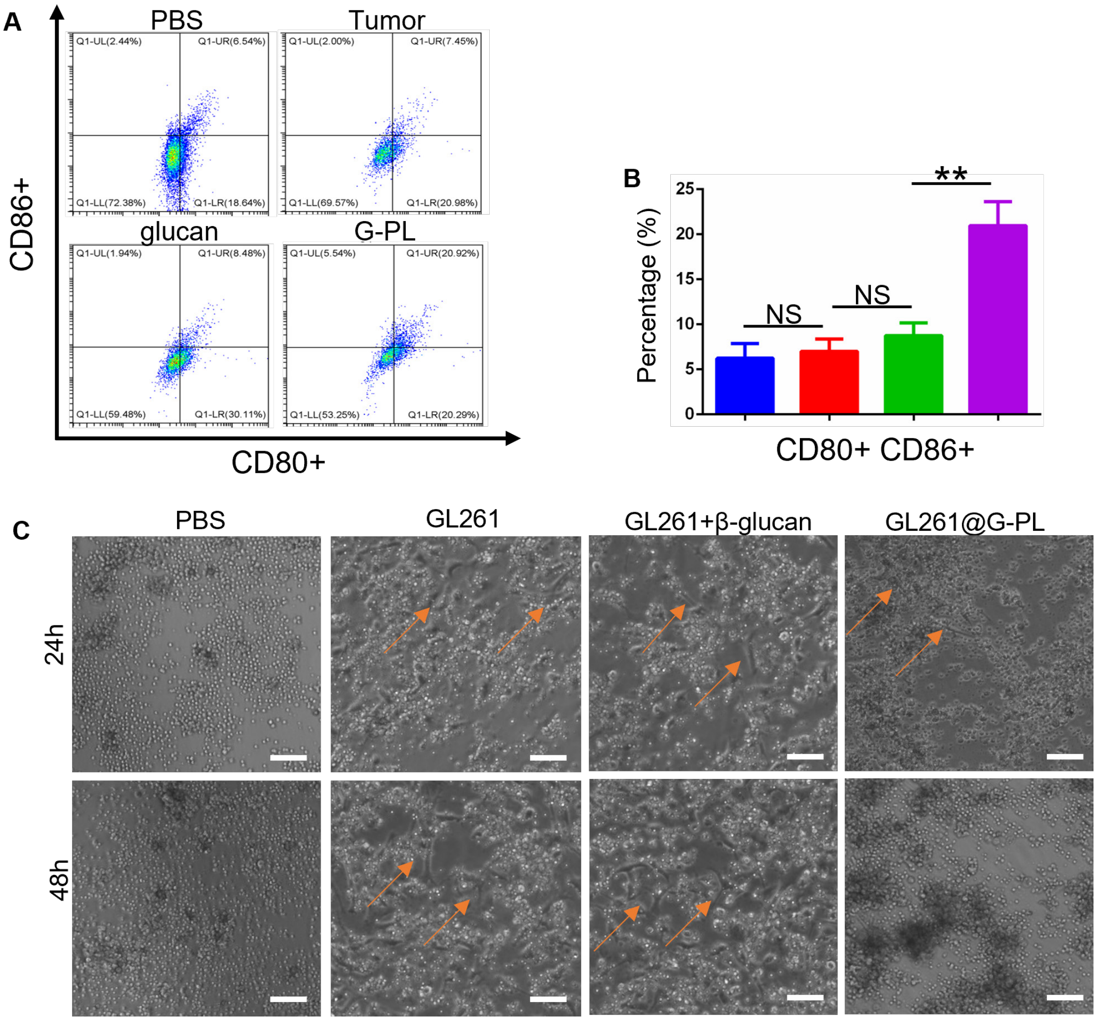
A
PBS
Tumor
glucan
G-PL
CD86+
CD80+
Q1-UL(2.44%)
Q1-UR(6.54%)
Q1-LL(72.38%)
Q1-LR(18.64%)
Q1-UL(2.00%)
Q1-UR(7.45%)
Q1-LL(69.57%)
Q1-LR(20.98%)
Q1-UL(1.94%)
Q1-UR(8.48%)
Q1-LL(59.48%)
Q1-LR(30.11%)
Q1-UL(5.54%)
Q1-UR(20.92%)
Q1-LL(53.25%)
Q1-LR(20.29%)
B
Percentage (%)
25
20
15
10
5
0
NS
NS
**
CD80+ CD86+
C
PBS
GL261
GL261+β-glucan
GL261@G-PL
24h
48h

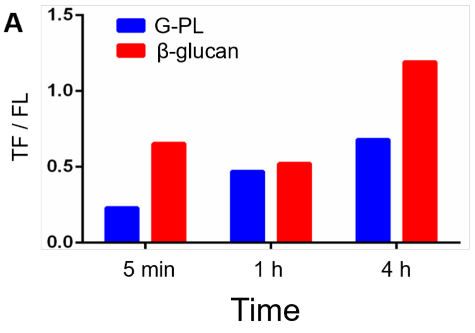
A
G-PL
β-glucan
TF / FL
1.5
1.0
0.5
0.0
5 min
1 h
4 h
Time

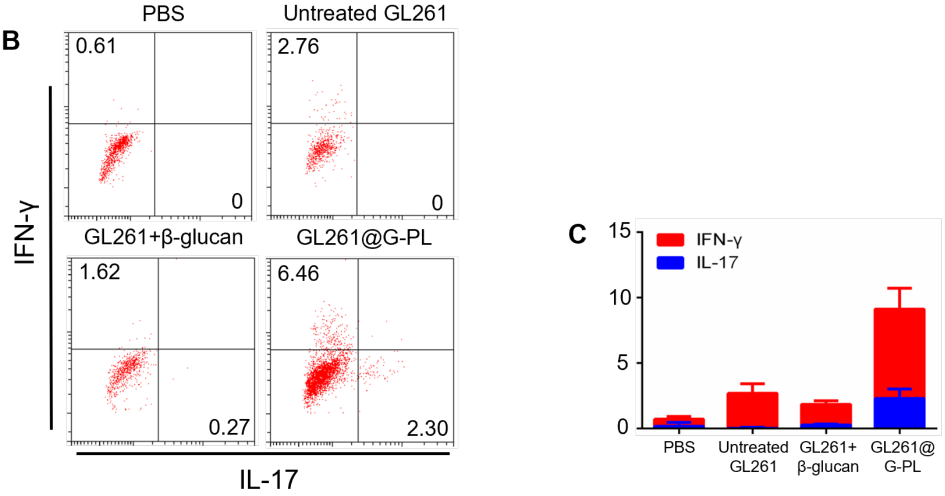
B
PBS
Untreated GL261
0.61
0
2.76
0
GL261+β-glucan
GL261@G-PL
1.62
0.27
6.46
2.30
IFN-γ
IL-17
C
IFN-γ
IL-17
15
10
5
0
PBS
Untreated GL261
GL261+ β-glucan
GL261@ G-PL

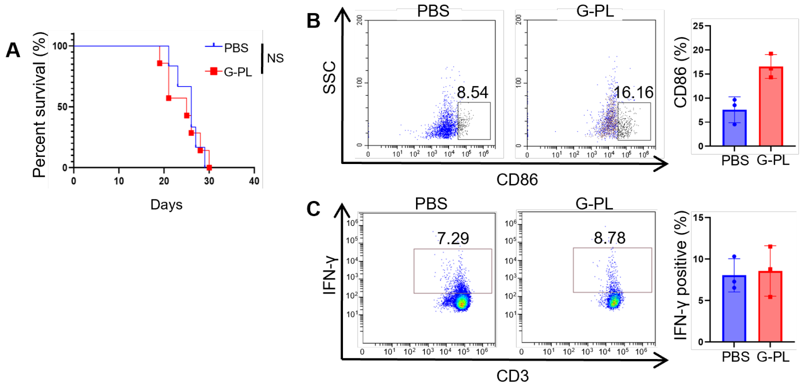
A
PBS
G-PL
NS
Percent survival (%)
Days
B
PBS
G-PL
SSC
8.54
16.16
CD86
CD86 (%)
PBS
G-PL
C
PBS
G-PL
IFN-γ
7.29
8.78
CD3
IFN-γ positive (%)
PBS
G-PL

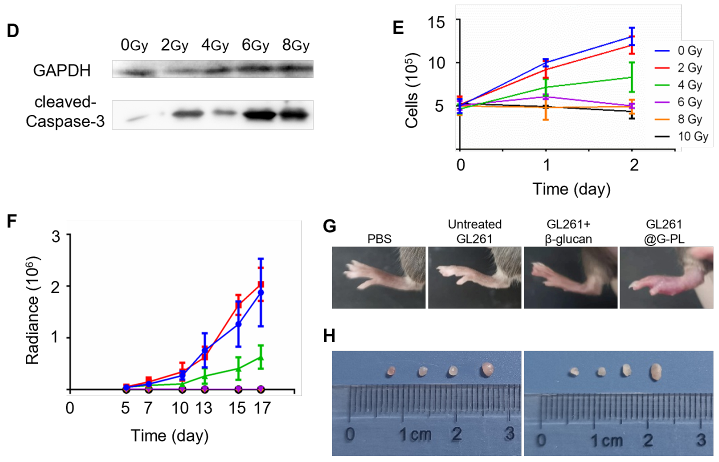
D
0Gy
2Gy
4Gy
6Gy
8Gy
GAPDH
cleaved-Caspase-3
E
Cells (10^5)
Time (day)
0 Gy
2 Gy
4 Gy
6 Gy
8 Gy
10 Gy
F
Radiance (10^6)
Time (day)
G
PBS
Untreated GL261
GL261+ β-glucan
GL261 @G-PL
H
1 cm

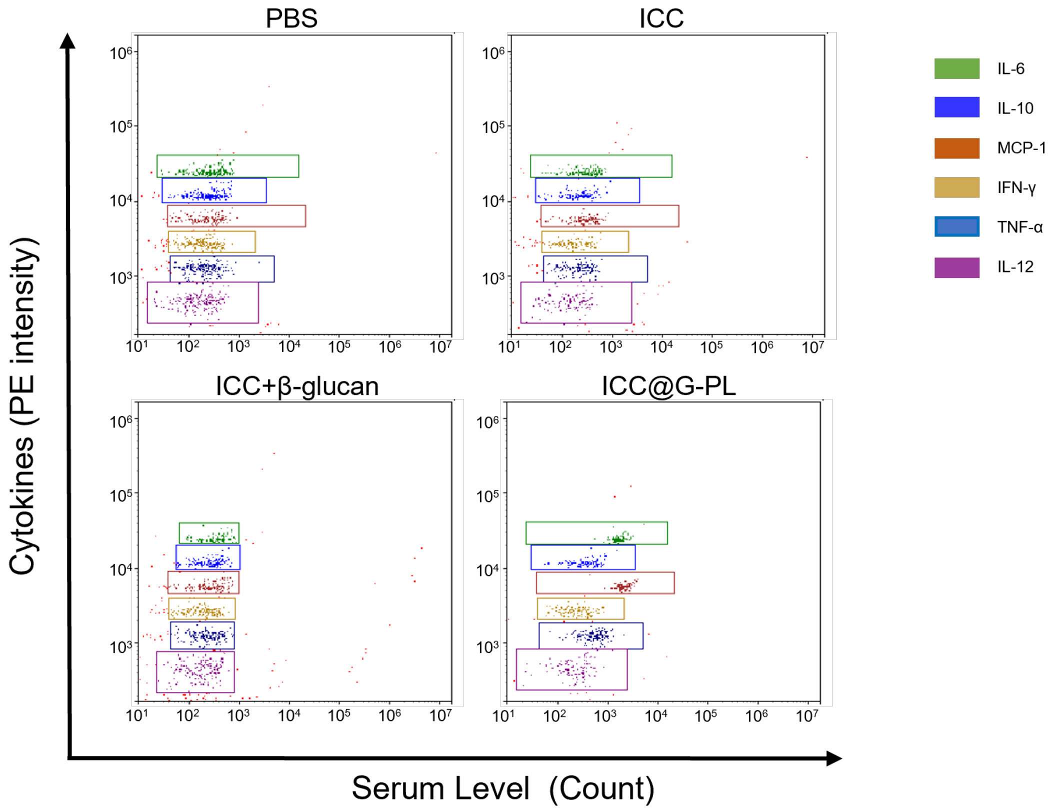

PBS
ICC
ICC+β-glucan
ICC@G-PL
Cytokines (PE intensity)
Serum Level (Count)
IL-6
IL-10
MCP-1
IFN-γ
TNF-α
IL-12

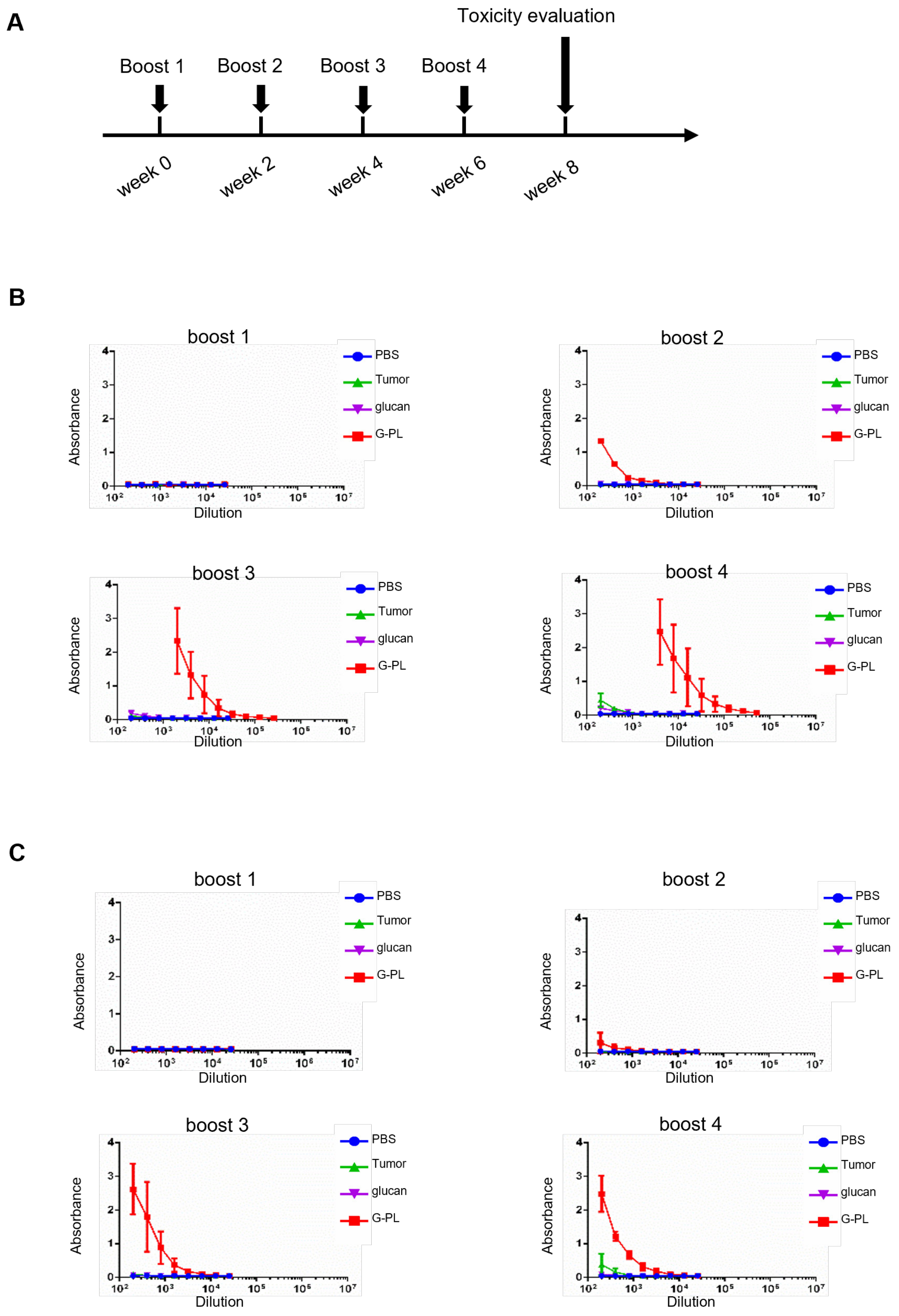
A
Toxicity evaluation
Boost 1
Boost 2
Boost 3
Boost 4
week 0
week 2
week 4
week 6
week 8
B
boost 1
boost 2
boost 3
boost 4
Absorbance
Dilution
PBS
Tumor
glucan
G-PL
C
boost 1
boost 2
boost 3
boost 4
Absorbance
Dilution
PBS
Tumor
glucan
G-PL

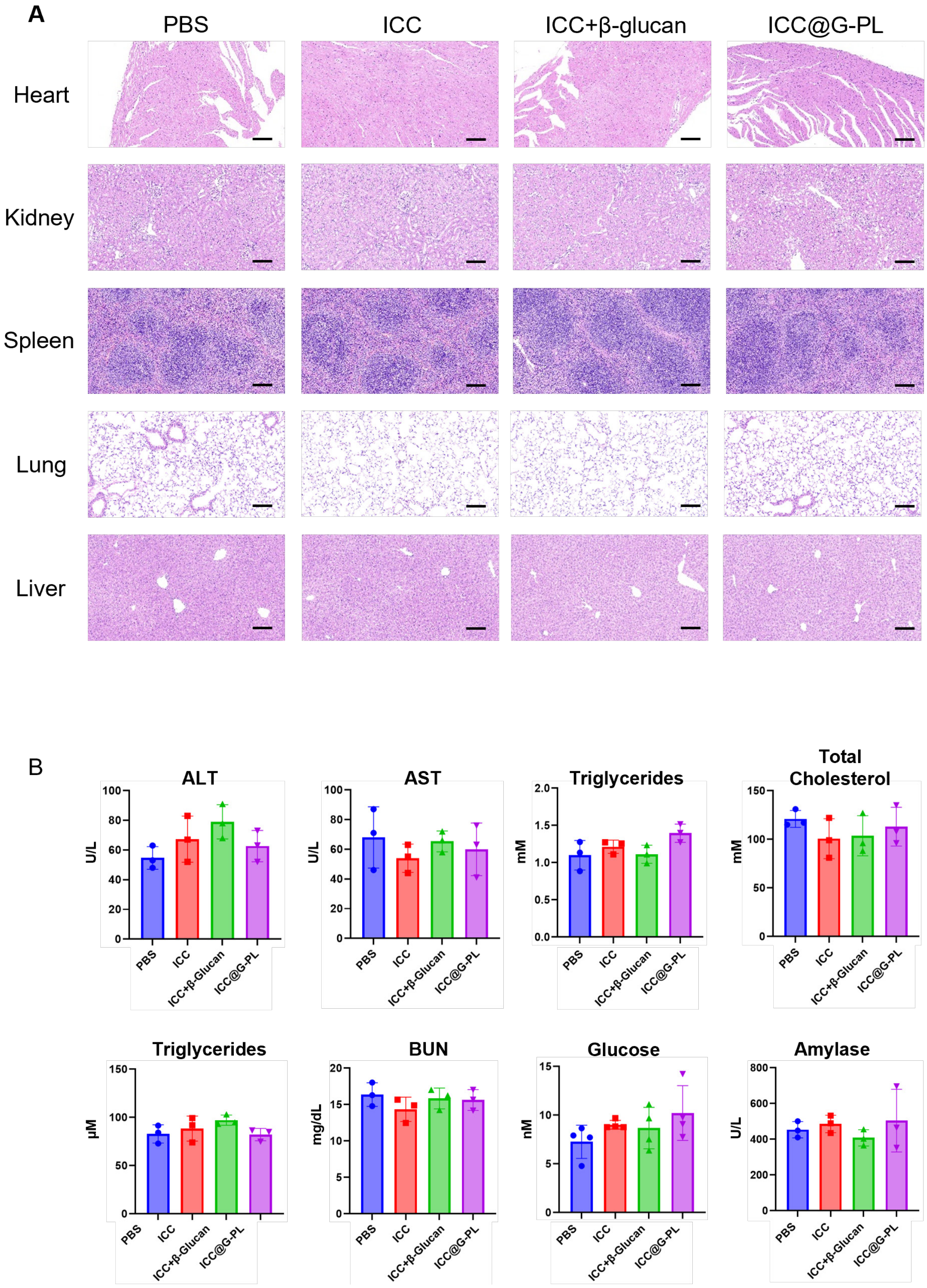

A
PBS
ICC
ICC+β-glucan
ICC@G-PL
Heart
Kidney
Spleen
Lung
Liver
B
ALT
AST
Triglycerides
Total Cholesterol
Triglycerides
BUN
Glucose
Amylase
U/L
mM
μM
mg/dL
nM
PBS
ICC
ICC+β-Glucan
ICC@G-PL

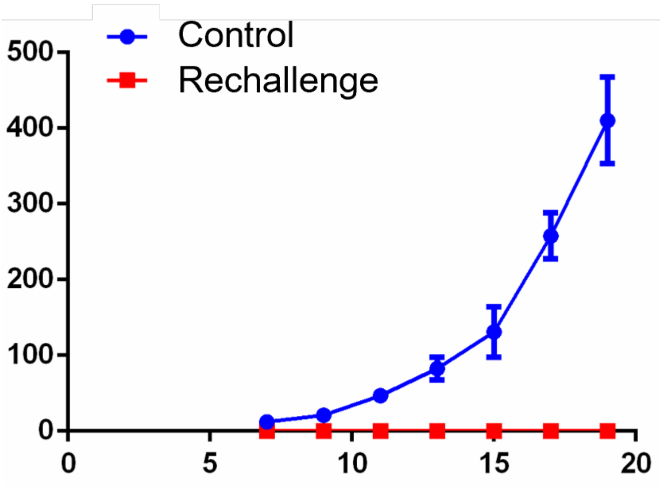
Control
Rechallenge
500
400
300
200
100
0
0
5
10
15
20

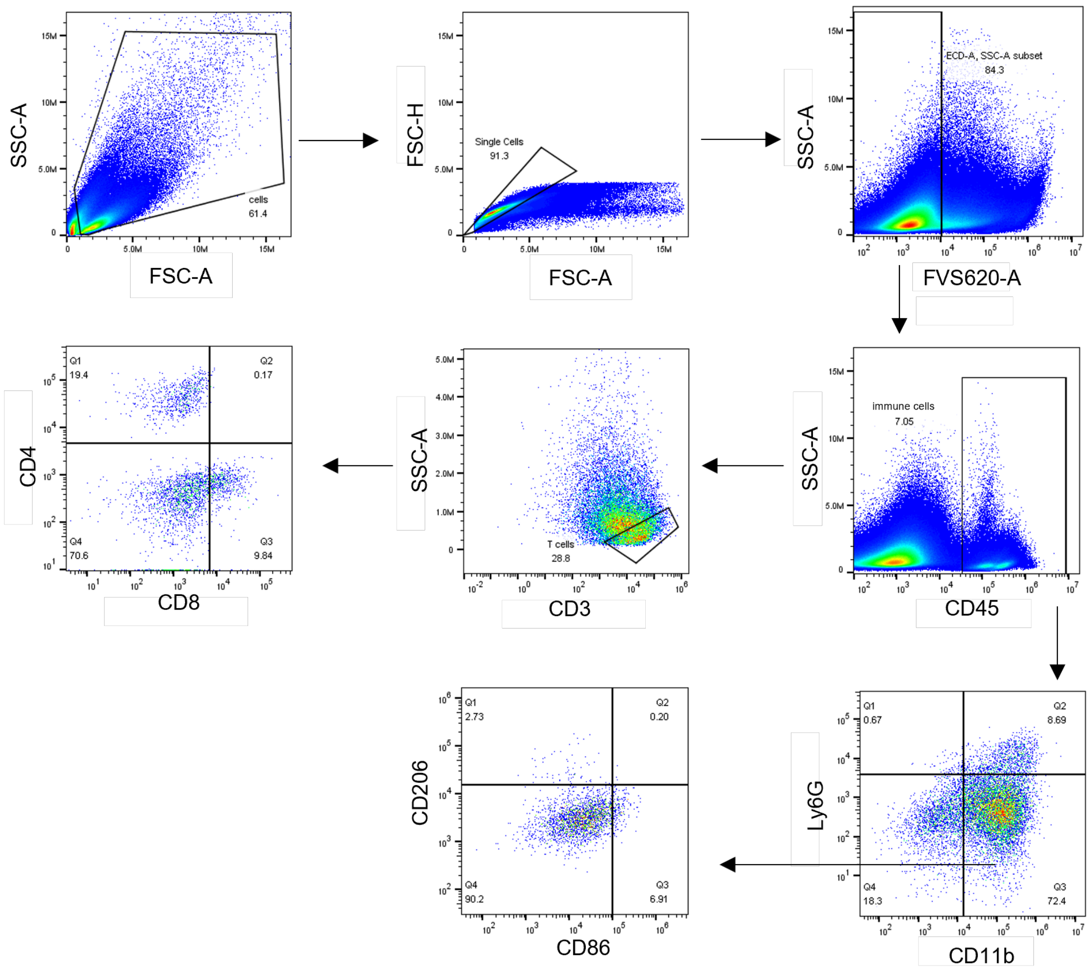
SSC-A
FSC-A
cells
61.4
FSC-H
FSC-A
Single Cells
91.3
SSC-A
FVS620-A
ECD-A, SSC-A subset
84.3
SSC-A
CD45
immune cells
7.05
SSC-A
CD3
T cells
28.8
CD4
CD8
Q1
19.4
Q2
0.17
Q3
9.84
Q4
70.6
Ly6G
CD11b
Q1
0.67
Q2
8.69
Q3
72.4
Q4
18.3
CD206
CD86
Q1
2.73
Q2
0.20
Q3
6.91
Q4
90.2
